# Racial and ethnic disparities in diagnosis and treatment outcomes among US-born people diagnosed with tuberculosis, 2003–19: an analysis of national surveillance data

**DOI:** 10.1016/S2468-2667(23)00276-1

**Published:** 2024-01

**Authors:** Mathilda Regan, Yunfei Li, Nicole A Swartwood, Terrika Barham, Garrett R Beeler Asay, Ted Cohen, Andrew N Hill, C Robert Horsburgh, Awal Khan, Suzanne M Marks, Ranell L Myles, Joshua A Salomon, Julie L Self, Nicolas A Menzies

**Affiliations:** Department of Global Health and Population, Harvard TH Chan School of Public Health, Boston, MA, USA; Department of Global Health and Population, Harvard TH Chan School of Public Health, Boston, MA, USA; Department of Global Health and Population, Harvard TH Chan School of Public Health, Boston, MA, USA; Office of Health Equity, National Center for HIV, Viral Hepatitis, STD, and TB prevention, US Centers for Disease Control and Prevention, Atlanta, GE, USA; Division of Tuberculosis Elimination, National Center for HIV, Viral Hepatitis, STD, and TB prevention, US Centers for Disease Control and Prevention, Atlanta, GE, USA; Yale School of Public Health, New Haven, CT, USA; Division of Tuberculosis Elimination, National Center for HIV, Viral Hepatitis, STD, and TB prevention, US Centers for Disease Control and Prevention, Atlanta, GE, USA; Departments of Epidemiology, Biostatistics, Global Health, and Medicine, Boston University Schools of Public Health and Medicine, Boston, MA, USA; Division of Tuberculosis Elimination, National Center for HIV, Viral Hepatitis, STD, and TB prevention, US Centers for Disease Control and Prevention, Atlanta, GE, USA; Division of Tuberculosis Elimination, National Center for HIV, Viral Hepatitis, STD, and TB prevention, US Centers for Disease Control and Prevention, Atlanta, GE, USA; Office of Health Equity, National Center for HIV, Viral Hepatitis, STD, and TB prevention, US Centers for Disease Control and Prevention, Atlanta, GE, USA; Department of Health Policy, Stanford University, Stanford, CA, USA; Division of Tuberculosis Elimination, National Center for HIV, Viral Hepatitis, STD, and TB prevention, US Centers for Disease Control and Prevention, Atlanta, GE, USA; Department of Global Health and Population, Harvard TH Chan School of Public Health, Boston, MA, USA

## Abstract

**Background:**

Persistent racial and ethnic disparities in tuberculosis incidence exist in the USA, however, less is known about disparities along the tuberculosis continuum of care. This study aimed to describe how race and ethnicity are associated with tuberculosis diagnosis and treatment outcomes.

**Methods:**

In this analysis of national surveillance data, we extracted data from the US National Tuberculosis Surveillance System on US-born patients with tuberculosis during 2003–19. To estimate the association between race and ethnicity and tuberculosis diagnosis (diagnosis after death, cavitation, and sputum smear positivity) and treatment outcomes (treatment for more than 12 months, treatment discontinuation, and death during treatment), we fitted log-binomial regression models adjusting for calendar year, sex, age category, and regional division. Race and ethnicity were defined based on US Census Bureau classification as White, Black, Hispanic, Asian, American Indian or Alaska Native, Native Hawaiian or Pacific Islander, and people of other ethnicities. We quantified racial and ethnic disparities as adjusted relative risks (aRRs) using non-Hispanic White people as the reference group. We also calculated the Index of Disparity as a summary measure that quantifies the dispersion in a given outcome across all racial and ethnic groups, relative to the population mean. We estimated time trends in each outcome to evaluate whether disparities were closing or widening.

**Findings:**

From 2003 to 2019, there were 72 809 US-born individuals diagnosed with tuberculosis disease of whom 72 369 (35·7% women and 64·3% men) could be included in analyses. We observed an overall higher risk of any adverse outcome (defined as diagnosis after death, treatment discontinuation, or death during treatment) for non-Hispanic Black people (aRR 1·27, 95% CI 1·22–1·32), Hispanic people (1·20, 1·14–1·27), and American Indian or Alaska Native people (1·24, 1·12–1·37), relative to non-Hispanic White people. The Index of Disparity for this summary outcome remained unchanged over the study period.

**Interpretation:**

This study, based on national surveillance data, indicates racial and ethnic disparaties among US-born tuberculosis patients along the tuberculosis continuum of care. Initiatives are needed to reduce diagnostic delays and improve treatment outcomes for US-born racially marginalised people in the USA.

**Funding:**

US Centers for Disease Control and Prevention.

## Introduction

In 2022, the USA reported 8300 cases of tuberculosis and an incidence of 2·5 cases per 100 000,^1^ exceeding the US Centers for Disease Control and Prevention (CDC)’s elimination goal of less than one case per million.^2^ Although tuberculosis incidence decreased by 79% in the USA during 1992–2020, progress towards elimination slowed between 2015 and 2020.^1^ One explanation for this trend is the increasing proportion of people with tuberculosis who were born in countries where there is a greater risk of tuberculosis exposure (currently more than 70% of cases); between 1993 and 2020 tuberculosis incidence decreased 90% among US-born people and 66% among non-US-born people. Additionally, although there has been an overall decrease in the risk of tuberculosis across racial and ethnic groups, differences between these groups have persisted. A study of tuberculosis incidence trends in the USA during 2003–16 found that incidence rate ratios increased or stayed the same over the study period for all racially marginalised people (both US-born and non-US-born people) compared with White people.^3^ A study that examined tuberculosis cases reported during 2001–19 for US-born individuals older than 50 years found that not only was the incidence significantly higher for racially marginalised people relative to White people, but these disparities appeared to be widening over time for some groups (American Indian or Alaska Native, and Native Hawaiian or Pacific Islander).^4^

Health disparities are preventable differences in health or opportunity to achieve optimal health, and are linked to economic, social, or environmental disadvantage.^5,6^ Racial and ethnic differences in the incidence and experience of tuberculosis result from a complex web of interrelated factors. These factors include the long-term effects of structural racism (such as racial segregation in housing), institutional racism (such as discriminatory policies and practices that have been embedded within a health facility), interpersonal racism (such as discrimination from health providers), internalised racism (such as adverse psychological outcomes because of the internalisation of racist ideology), and differences in the prevalence of comorbidities such as HIV, which weaken immune function.^7,8^ Consequences of racism and discrimination include disparities in socioeconomic status, access to health care, inclusion in public health surveillance, the built environment, and psychosocial stress.^5,6,7^ Although outpatient treatment is covered financially by public health departments in the USA regardless of insurance status, hospital admission, which is needed for approximately half of patients with tuberculosis,^9^ is not. Further, even for insured patients, the costs associated with hospital admission are substantial.^9^

The tuberculosis continuum of care is a framework to track patient retention in sequential stages of care, from their first interaction with the health-care system to the final treatment success (or failure).^10^ Evidence from outcomes along the care continuum can measure progress towards reducing differences between patient groups and reveal opportunities for targeted interventions. This study aimed to describe the association between race and ethnicity and tuberculosis diagnosis (diagnosis after death, cavitation, and sputum smear positivity) and treatment (treatment for more than 12 months, treatment discontinuation, and death during treatment) outcomes among US-born people in 2003–19, using US tuberculosis surveillance data. To the best of our knowledge, this is the first study examining racial and ethnic disparities in diagnosis and treatment outcomes among US-born people.

## Methods

### Tuberculosis data and covariates

We extracted individual-level data from the National Tuberculosis Surveillance System (NTSS) on US-born individuals diagnosed with tuberculosis disease during 2003–19. All tuberculosis cases in the USA are required to be reported to the NTSS and are verified according to specified laboratory and clinical criteria.^1^ We defined being born in the USA as being born in any of the 50 US states or the District of Columbia as well as individuals born abroad to a US citizen or born in a US-affiliated jurisdiction, following US Census definitions. We excluded individuals with multidrug-resistant tuberculosis (0·40%), for whom the criteria for treatment success differs from individuals without multidrug-resistant tuberculosis.^11^ We also excluded people who had missing data on race and ethnicity (0·23%). In addition to variables on tuberculosis diagnosis and treatment outcomes (described hereafter), we extracted data on patient sex at birth (the NTSS does not collect data on gender), race and ethnicity, age, and county. Data on racial and ethnic identity was self-reported and we used the following categories on the basis of US Census Bureau single-race classification:^12^ non-Hispanic White; non-Hispanic Black; Hispanic; non-Hispanic Asian, including individuals who identify as originating from the Far East, southeast Asia, or south Asia; non-Hispanic American Indian or Alaska Native; non-Hispanic Native Hawaiian or Pacific Islander; or non-Hispanic of other ethnicity (hereafter referred to as White, Black, Hispanic, Asian, American Indian or Alaska Native, Native Hawaiian or Pacific Islander, and people of other ethnicities).^12^ Because of the small number of people identifying as both Hispanic and non-White, we did not disaggregate racial and ethnic identities among Hispanic people for the main analysis. We categorised age as follows: younger than 1 year; 1–4 years; 5–14 years; 15–24 years; 25–34 years; 35–44 years; 45–54 years; 55–64 years; 65–74 years; 75–84 years; and older than or equal to 85 years. We used the state of report to create a location variable on the basis of the nine US Census Bureau regional divisions: Pacific; Mountain; West North Central; West South Central; East North Central; East South Central; South Atlantic; Middle Atlantic; and New England. Ethical approval and individual consent were not required because these data were collected as part of routine public health surveillance.

### Diagnosis outcomes

We analysed three indicators of disease severity at diagnosis, as measures of potential delayed tuberculosis diagnosis, as follows: diagnosis after death; presence of lung cavitation; and acid-fast-bacilli (AFB)-positive sputum smear microscopy results. We included all causes of death for people who were diagnosed with tuberculosis after death, as previous studies have shown that death certificates often misclassify deaths caused by tuberculosis.^13^ We classified cavitation and smear positivity as indicative of more severe disease at case presentation on the basis of natural history studies that show the risks of smear positivity and cavitation increase as the disease progresses, and that patients with cavitation or smear positivity have a poorer prognosis.^14,15^ Lung cavitation was captured by a binary variable (presence or absence of cavitation) only available for individuals with pulmonary tuberculosis and a recorded abnormal radiograph or CT scan result. AFB sputum smear positivity was captured by a binary variable (smear positive or smear negative) only available for individuals with a recorded AFB sputum smear result. We excluded people with concomitant extrapulmonary and pulmonary tuberculosis (8·8%) from our analysis of cavitation and sputum smear positivity because the presentation of disease differs in this subgroup (eg, patients who have advanced extrapulmonary disease with concomitant pulmonary disease might not present with cavitation or smear positivity).

### Treatment outcomes

We analysed three indicators of tuberculosis treatment outcome: treatment duration longer than 12 months; treatment discontinuation; and death during treatment. Treatment that lasted longer than 12 months was derived from data on the length of therapy in days. For the outcome of treatment of more than 12 months only, we excluded people who were not eligible to complete treatment in 12 months, people who required extended treatment because of clinical reasons, rifampin resistance, or an adverse drug reaction (collectively 1·8%), and people who died (8·5%) or were lost to follow-up before 12 months (2·5%). This outcome reflects the US Centers for Disease Control 2025 National Performance Target that treatment should not exceed 12 months for 95% of people who are eligible to complete treatment within 12 months.^16^ Treatment discontinuation included any patient who stopped treatment because of refusal of treatment (n=629), or who could not be located by the health department before the completion of treatment (n=1181). For the treatment discontinuation and death during treatment outcomes, we excluded people who stopped treatment because of an adverse drug reaction (0·16%) or unspecified other reasons (0·80%), and people who were lost to follow-up because of moving (0·40%; this option was removed as a valid option and recategorised as other beginning in 2009). Death during treatment included any death that occurred after diagnosis but before treatment completion.

### Summary outcome

As a summary of outcomes along the care continuum, we estimated a composite outcome describing whether an individual had any outcome precluding successful treatment completion, defined as any of diagnosis after death, treatment discontinuation, or death during treatment.

### Statistical analysis

For each of the seven outcomes (diagnosis after death, cavitation, smear positive, treatment for more than 12 months, treatment discontinuation, death during treatment, or the summary outcome), we fitted log-binomial regression models adjusting for sex, age category, calendar year, and regional division, to estimate relative risks for each racial and ethnicity category, using people who are White as the reference category because previous studies have reported the lowest rates of tuberculosis among this group.^3,4^ In a second set of models, we included an interaction term between race and ethnicity and calendar year, to assess time trends in relative risks.

In addition to these relative risks, we calculated a weighted version of the Index of Disparity^17^ for each outcome using the formula

$$Index\;of\;Disparity=100*\left(\sum_{j=1}^{J}\;\left|r_{j}-R\right|*w_{j}\right)/R$$

The Index of Disparity that we used measures the average proportional deviation from the mean value of the outcome across all groups and is presented as a percentage. In this formula, $r_{j}$ is the value of an outcome for a given population subgroup, R is the mean value of the outcome for the overall population, and $w_{j}$ is the proportion of each group’s population share within the overall population. For this analysis we operationalized $r_{j}$ and R as the mean value of each study outcome (eg, diagnosis after death) for each racial and ethnic group, when standardized to the distribution of sex, age category, calendar year, and regional division in the overall study population using predicted probabilities from the regression models. We adopted this approach to control for the effect of these factors when estimating results for the Index of Disparity.

We used a Monte Carlo approach to propagate the uncertainty in analytic outcomes, simulating 1000 random parameter values from the fitted coefficient estimates of the regression models (via the mvrnorm function in the MASS package),^18^ assuming a multivariate normal distribution. All analyses were carried out in R (version 4.2.0).^19^

### Sensitivity analysis

We did several sensitivity analyses to evaluate the robustness of our results. First, we re-estimated our baseline models with two alternative US Census Bureau location specifications, four regional divisions (northeast, midwest, south, and west) and six urban and rural designations (large central metro, large fringe metro, medium metro, small metro, micropolitan, and non-core [rural]). Second, we re-estimated the analysis of time trends using dummy variables for each calendar year, as four categorial time periods (2003–06, 2007–10, 2011–14, 2015–19), with a cubic spline, and as a random effect in a linear-mixed model. We also re-ran our models for cavitation and AFB-positive sputum smear with two alternative specifications, which were first excluding individuals who received a positive HIV laboratory test, and second including only individuals with a negative HIV laboratory test. We re-estimated our baseline models to include the 292 people with multidrug-resistant-tuberculosis. We examined racial and ethnicity subgroups among people identifying as Hispanic (Hispanic White, Hispanic Black, and Hispanic of other ethnicity), using non-Hispanic White as the reference group.

### Role of the funding source

Employees of the funder participated as coauthors on the study, contributing to study design, data analysis, data interpretation, manuscript preparation, and the decision to submit for publication.

## Results

From 2003 to 2019 (inclusive), 72 809 US-born individuals were diagnosed with tuberculosis disease (appendix p 2). After excluding the 292 individuals with multidrug-resistant tuberculosis and the 148 with missing race and ethnicity data, 72 369 cases remained in our analytic sample (appendix p 2). The sample included 23 822 (33·0%) White, 30 198 (41·7%) Black, 13 181 (18·2%) Hispanic, 2268 (3·1%) Asian, 2176 (3·0%) American Indian or Alaska Native, 425 (0·6%) Native Hawaiian or Pacific Islander people, and 299 (0·4%) people of other ethnicities. The proportion of women ranged from 32·0% in those with White ethnicity to 45·0% in Native Hawaiian or Pacific Islander people. The percentage of individuals 65 years and older ranged from 36·7% among people with White ethnicity to 10·5% among people with Asian ethnicity (appendix p 1).

The association between race and ethnicity and three diagnosis outcomes, comprising diagnosis after death, presence of lung cavitation, and AFB-positive sputum smear microscopy are presented in table 1. 2397 (3·3%) of 72 369 people received a postmortem diagnosis of tuberculosis disease (appendix p 2). After adjusting for age, calendar year, sex, and location, Black and Hispanic people had a greater risk of diagnosis after death than White people. Among the 51 572 people with pulmonary tuberculosis with a recorded abnormal chest radiograph or CT scan result, 16 729 (32·4%) had evidence of cavitation (appendix p 2). Black people with pulmonary tuberculosis had a greater adjusted risk of cavitation at diagnosis than White people, whereas Asian people had a lower risk. More than half of the 45 322 people with pulmonary tuberculosis who received sputum smear microscopy tested AFB positive (25 510 [56·3%]; appendix p 2). Compared with White people, we observed a higher adjusted risk of AFB smear positivity at diagnosis among Black people, Hispanic people, and American Indian or Alaska Native people.

Associations between race and ethnicity and three treatment outcome measures (treatment >12 months, treatment discontinuation, and death during treatment) are presented in table 2. Of the 69 941 people who were alive at diagnosis, 60 121 (86·0%) completed treatment and 52 522 (75·1%) completed treatment within 12 months. 6438 (9·2%) people took longer than 12 months to complete treatment for reasons other than a clinical recommendation, and 6451 (9·2%) people died during treatment (appendix p 2). After adjusting for age, calendar year, sex, and location, Black people, Hispanic people, and American Indian or Alaska Native people had greater risks of a treatment duration over 12 months, relative to White people. Black people and Hispanic people also had higher risks of treatment discontinuation. Asian people had lower risks of treatment discontinuation than White people. Black, Hispanic, and American Indian or Alaska Native people had a higher risk of dying during treatment than White people. Asian people did not have a significantly higher risk of death during treatment than White people.

**Figure 1:**
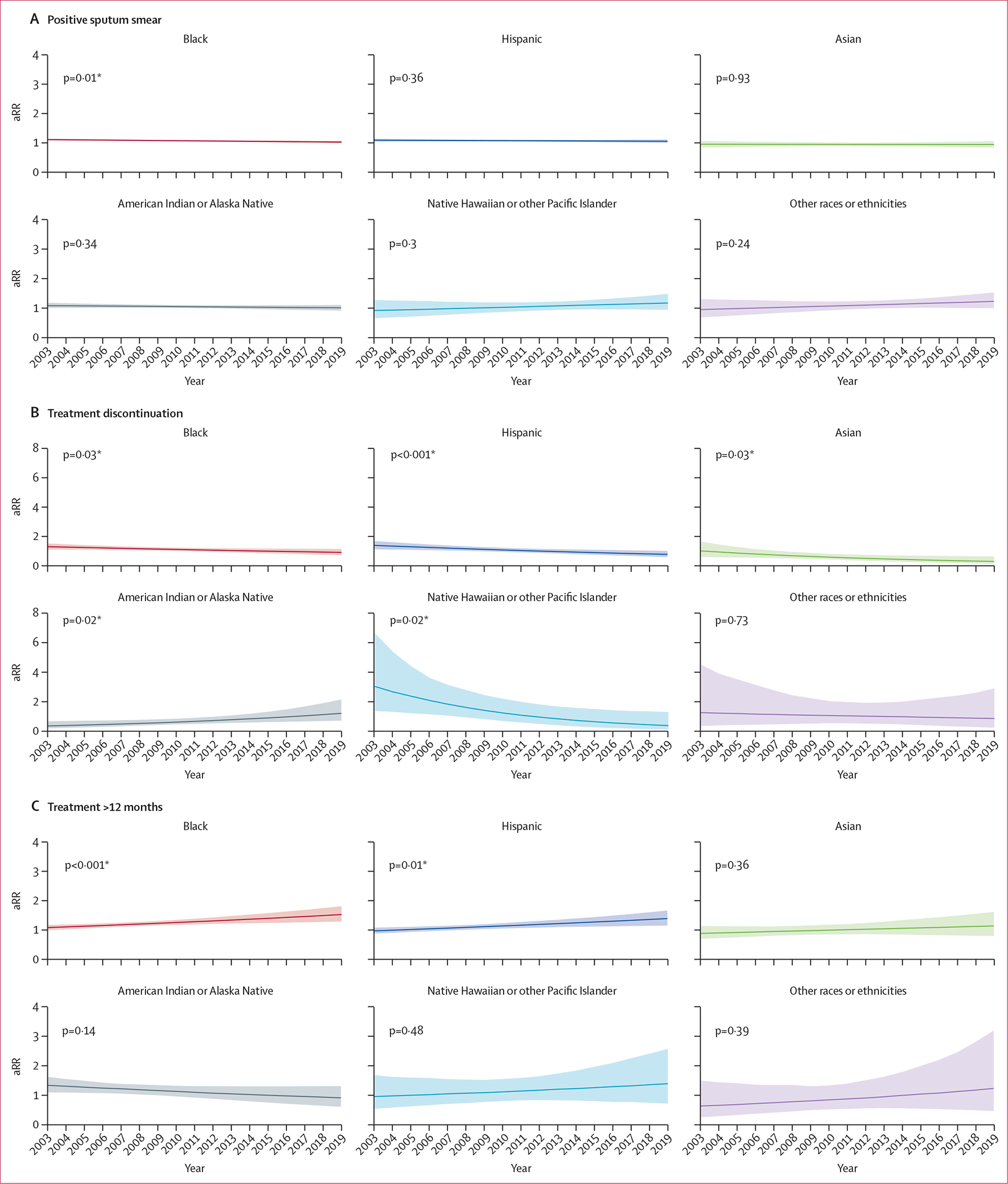
Estimated risk of select tuberculosis outcomes over time relative to White people, adjusted for sex, age category, geographical region, and year Solid line indicates point estimates and shaded region indicates 95% CIs. Sputum-smear positive estimates are restricted to individuals with pulmonary tuberculosis and a recorded AFB sputum smear result. p values are for the interaction between years. (A) People who tested positive on sputum smear. (B) People who discontinued treatment. (C) People who had treatment for more than 12 months. AFB=acid-fast bacilli. aRR=adjusted relative risk.

Time trends in relative risks for each racial or ethnic group compared with White people for select outcomes (smear positive, treatment over 12 months, and treatment discontinuation) are shown in figure 1 (time trends for the remaining outcomes are shown in the appendix pp 4–5). During 2003–19, the adjusted relative risk of having a positive sputum smear at diagnosis decreased by 7·3% (95% CI 1·8–12·4) for Black people. We observed an increase of 37·9% (12·9–65·7) in the relative risk of treatment duration exceeding 12 months for Black people and an increase of 39·2% (10·0–75·7) for Hispanic people. Risk of treatment discontinuation relative to White people increased among American Indian or Alaska Native people by 223·9% (18·3–835·3). We did not observe statistically significant changes in the risk of diagnosis after death, cavitation at diagnosis, or death during treatment for any population over this time period. Time trends in adjusted predicted probabilities for each of the six racial or ethnicity categories are shown in the appendix (pp 12–14).

**Figure 2:**
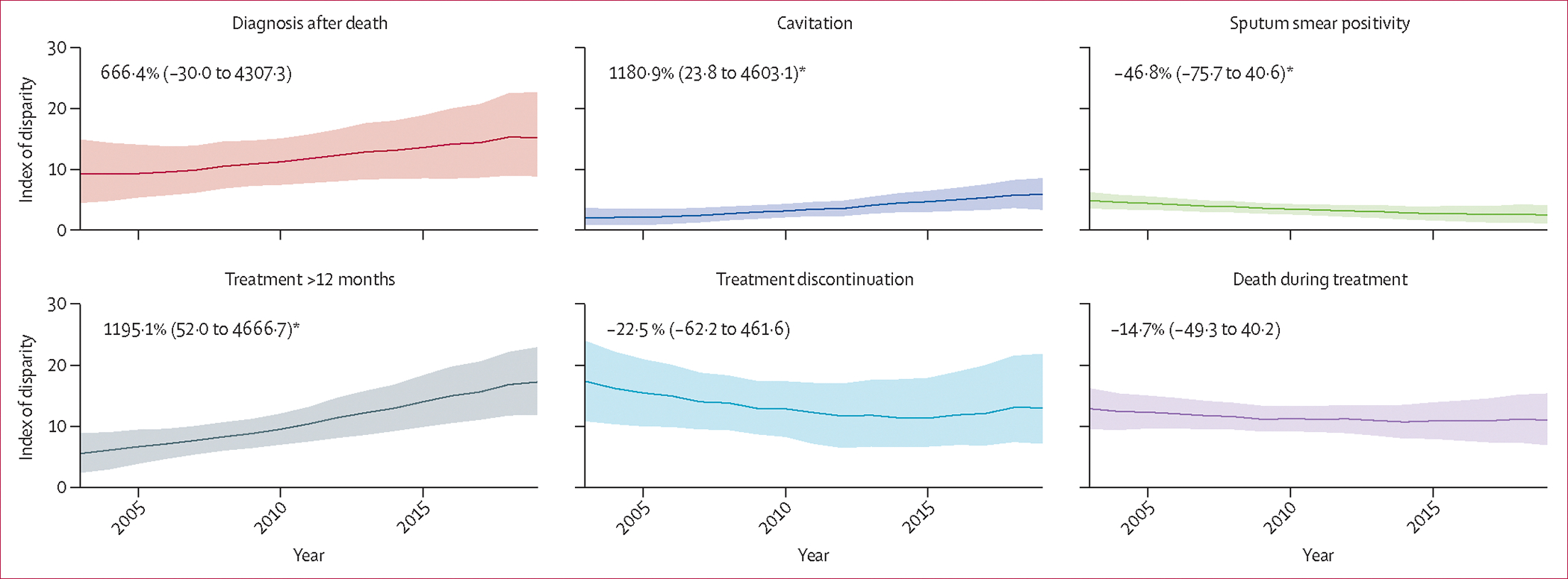
Estimated index of disparity over time for case presentation and treatment outcomes Solid line indicates point estimates and shaded region indicates 95% CIs. Cavitation estimates are restricted to people with pulmonary tuberculosis and a recorded abnormal radiograph or CT scan result. Sputum-smear positive estimates are restricted to individuals with pulmonary tuberculosis and a recorded AFB sputum smear result. Data are percentage change (95% CI). AFB=acid-fast bacilli. *p<0·05.

Trends in the Index of Disparity over time for each of the six outcomes are presented in figure 2. Across the six outcomes we observed the highest disparity in 2019 for treatment that spanned more than 12 months (Index of Disparity 16·70, 95% CI 11·13–22·23), followed by diagnosis after death (Index of Disparity 15·54, 8·68–22·41), and treatment discontinuation (Index of Disparity 13·14, 7·82–20·22). During 2003–19, we observed a statistically significant (p<0·05) decrease of 46·8% (95% CI 0·6 to 75·7) in the Index of Disparity for having a positive sputum smear. We also observed a statistically significant increase (p<0·05) in the Index of Disparity between 2003 and 2019 for cavitation and treatment over 12 months.

We observed an increased risk of any adverse outcome (defined as diagnosis after death, treatment discontinuation, or death during treatment) for Black (aRR 1·27, 95% CI 1·22–1·32), Hispanic (aRR 1·20, 1·14–1·27), and American Indian or Alaska Native people (aRR 1·24, 1·12–1·37). We did not observe a significant change over time in risk of the summary outcome relative to White people for any group.

In sensitivity analyses that changed the geographical specification, time specification, and model specification (cubic spline, random effects), we did not observe qualitatively different results (appendix pp 6–11). Additionally, including the 292 patients with multidrug-resistant tuberculosis in the overall study population and excluding patients with HIV for the cavitation and smear-positive outcomes had little effect on the results (appendix pp 7–8). In the analysis of Hispanic racial or ethnicity subgroups, we observed that people who identified as Hispanic and Black had a greater risk of death during treatment relative to those who identified as non-Hispanic White (aRR 1·67, 95% CI 1·28–2·09). Incidence rates were lower for Hispanic and White people, but still elevated relative to non-Hispanic White people (aRR 1·18, 1·09–1·28) (appendix p 18).

## Discussion

In this study we quantified racial and ethnic disparities among US-born patients with tuberculosis along the continuum of care for tuberculosis disease in the USA, and we evaluated how these disparities changed during 2003–19. Over the study period the total number of tuberculosis deaths (either before diagnosis or during treatment) among US-born people decreased steadily during 2003–19, mirroring the decrease in tuberculosis incidence that occurred over the same period.^1^ However, disparities between racial and ethnic groups have persisted, as described by elevated risks of being diagnosed after death, more severe case presentation at diagnosis, dying before diagnosis or during treatment, and treatment discontinuation for racially marginalised people compared with people who are White. Overall, people who are Black, Hispanic, and American Indian or Alaska Native had a higher risk of an adverse tuberculosis outcome (defined as death either before diagnosis or during treatment, or treatment discontinuation) than White people.

Our findings suggest that, although prevention and treatment efforts (such as increases in directly observed therapy over time)^1^ have improved tuberculosis outcomes among US-born people, targeted initiatives might be needed to ensure early detection of tuberculosis, particularly among racially marginalised people. Although the raw percentages for diagnosis after death and death during treatment were higher for White people, after adjusting for covariates of age, sex, year, and region, the risk of these outcomes was higher for Black and Hispanic people. We observed a modestly higher risk of cavitation among patients who are Black, and a modestly higher risk of smear-positive results among patients who are Black, Hispanic, and Native American or Alaska Native than people who are White. The Index of Disparity increased between 2003 and 2019 for cavitation. Evidence suggests that increased access to newer diagnostic technology such as Nucleic Acid Amplification Testing (NAAT) would reduce time to treatment.^20,21^ Although use of NAATs has increased in the past decade, there is still opportunity for expansion; a recent study of surveillance data found that nearly half of cases reported during 2011–17 did not use a NAAT.^21^ Disparities in access to diagnostic technology such as NAAT or radioagraphy for pulmonary tuberculosis is an important area for future research.

Previous studies that have looked at disparities in tuberculosis mortality before treatment^14,15,22,23^ found that people with HIV and those with more extensive disease had a higher risk of dying before diagnosis. However, increased access to antiretroviral treatment has led to improved health outcomes for people with tuberculosis and HIV coinfection and a lower risk of tuberculosis disease for people with HIV.^22,23^ In our sensitivity analysis for risk of cavitation, excluding people with HIV did not change our results, suggesting that the disparities in cavitation are caused by other factors.

A recent study of diagnostic delays using administrative claims among people who are privately insured in the USA reported a median of 24 days (IQR 10–45) between the first visit for tuberculosis symptoms and initiation of anti-tuberculosis treatment.^24^ In analyses within one US state, delays were longer among people who were older and immunocompromised, possibly because of the heterogeneity of clinical case presentation among these people and the presence of comorbidities.^25^ Although tuberculosis diagnosis and treatment has largely been managed by public health departments, private-sector tuberculosis care has become increasingly more common since the 1990s, particularly as the Affordable Care Act has increased access to health insurance.^26^ There is evidence that private providers are less likely to adhere to tuberculosis treatment guidelines than public providers.^27^ Increased collaboration between private providers and public health departments with tuberculosis expertise is needed.

We observed pervasive racial and ethnic disparities in treatment outcomes. We found that during 2003–19 the Index of Disparity for treatment exceeding 12 months increased, and the risk for Black and Hispanic people increased over this period relative to White people. Across all groups, the overall percentage of eligible people who completed treatment in 12 months fell short of the CDC target of 95%.^16^ Over time, the risk of treatment discontinuation increased for American Indian or Alaska Native people relative to White people but decreased for people in all other racial and ethnicity categories. Time trends in death during treatment were not significant, but overall Black, Hispanic, and American Indian or Alaska Native people had an elevated risk of death during treatment, relative to White people. Previous studies on risk factors for death during treatment in the USA found associations with HIV infection and severity of case presentation.^28^ A systematic review of death during and after treatment identified delays in presentation to the health system with initial symptoms as an important risk factor^28^ and this might also underlie the results observed in our study.

This study has several limitations. First, the analysis was restricted to individuals who made contact with the health system. Therefore, we might have excluded people living with tuberculosis who were unable to access care, which might be more common among racially marginalised people. Some of these individuals are captured by the diagnosis after death outcome but others probably died without being diagnosed. Additionally, we assessed severity of case presentation at diagnosis through cavitation and smear positivity as a proxy for access to timely care; however, differences in disease presentation by pulmonary or extrapulmonary disease site were not evaluated. Second, because of the small number of cases for people identifying as Native Hawaiian or Pacific Islander and people of other ethnicities, results for these populations should be interpreted with caution. Third, it is possible that we introduced selection bias because of the exclusion of people with missing race and ethnicity or outcome data. Fourth, although there is a programme in place to coordinate treatment continuation between jurisdictions, it is possible that some people who were classified as being lost to follow-up resumed treatment in a different jurisdiction. Finally, our results were restricted to US-born people; future work will need to investigate such disparaties in non-US-born tuberculosis patients.

Further research is needed to elucidate the mechanisms through which people who are Black, Hispanic, and American Indian or Alaska Native have a greater risk of adverse tuberculosis care outcomes. One study found that socioeconomic status explains approximately half of racial and ethnic disparities in tuberculosis incidence.^29^ Comorbidities that are associated with poorer tuberculosis outcomes, including HIV, diabetes, and renal disease disproportionally affect some racially marginalised populations.^7,8,28^ Additionally, there are substantial racial and ethnic disparities in health insurance coverage,^30^ access to timely care,^31^ and quality of health services in the USA.^32^ Differences in prevalence of risk factors (eg, lower prevalence of HIV, homelessness, and injection drug use) might also underlie our finding that Asian people had a lower risk of diagnosis after death, cavitation, and treatment discontinuation, than White people. We did not adjust for factors such as HIV status or socioeconomic status in this analysis, because controlling for these variables could obscure mechanisms through which these disparities exist. For example, low socioeconomic status might be part of the pathway through which racial segregation affects these outcomes. Statistical methods such as mediation analysis can help identify these potential pathways^33^ and inform future interventions. Further research is also needed on disparities in sequelae after tuberculosis disease; the disparities we observed in treatment outcomes might have led to a higher burden of lung disease after tuberculosis among these populations.^34^ There is scarce evidence from tuberculosis interventions that have measured changes in racial and ethnic disparities (apart from increased access to antiretroviral treatment). A community-based participatory intervention in the rural southeastern USA reduced overall tuberculosis incidence but did not reduce racial and ethnic disparities.^35^ Previous research has highlighted the importance of understanding how an individual’s experience of tuberculosis is informed by their sociocultural and historical context.^36^ The consideration of sociocultural factors such as social stigma alongside other socioenvironmental barriers is key to creating an enabling environment for diagnosis and treatment adherence. Future interventions to reduce racial and ethnic disparities in tuberculosis care should engage communities in their development, implementation, and evaluation to ensure that they are culturally appropriate, sensitive, and targeted at key drivers of disease outcomes.

Our analysis underscores the importance of identifying cases early in populations who have a high risk of tuberculosis disease and ensuring that these individuals have access to timely, appropriate care, to prevent disparities in tuberculosis diagnosis and treatment outcomes from persisting or increasing.

## Supplementary Material

Appendix

## Figures and Tables

**Table 1:** Tuberculosis case presentation at diagnosis by race and ethnicity among US-born people diagnosed with tuberculosis, USA 2003–19

	Diagnosis after death*		Cavitation†		Smear positive‡	
			
	cases/N (%)	aRR§ (95% CI)	cases/N (%)	aRR§ (95% CI)	cases/N (%)	aRR§ (95% CI)

White	945/23 812 (4·0%)	Ref	5888/18 114 (32·5%)	Ref	8895/16 425 (54·2%)	Ref
Black	1023/30 180 (3·4%)	1·22 (1·12–1·33)	7519/21 039 (35·7%)	1·03 (1·01–1·06)	11 308/19 214 (58·9%)	1·08 (1·06–1·10)
Hispanic	320/13 179 (2·4%)	1·30 (1·14–1·47)	2411/8853 (27·2%)	0·96 (0·92–1·00)	3779/6782 (55·7%)	1·08 (1·05–1·10)
Asian	23/2268 (1·0%)	0·74 (0·48–1·09)	289/1538 (18·8%)	0·82 (0·74–0·91)	516/1100 (46·9%)	0·95 (0·89–1·01)
American Indian or Alaska Native	71/2175 (3·3%)	1·09 (0·85–1·39)	504/1579 (31·9%)	1·05 (0·97–1·13)	821/1463 (56·1%)	1·06 (1·01–1·11)
Native Hawaiian or other Pacific Islander	9/425 (2·1%)	1·54 (0·75–2·75)	47/245 (19·2%)	0·88 (0·68–1·10)	87/161 (54·0%)	1·08 (0·93–1·22)
Other ethnicities	6/299 (2·0%)	0·88 (0·35–1·75)	71/204 (27·0%)	1·17 (0·97–1·37)	104/177 (58·8%)	1·12 (0·98–1·25)

Results exclude non-US-born patients and patients diagnosed with multidrug-resistant tuberculosis.

* Includes all causes of death and all sites of tuberculosis disease.

† Lung cavitation among patients with pulmonary tuberculosis who had an abnormal radiograph or CT scan (n=51 752).

‡ Positive sputum smear results among patients with pulmonary tuberculosis who had a sputum smear test result (n=45 322).

§ Adjusted relative risks adjusting for sex, categorical age (<1 year, 1–4 years, 5–14 years, 15–24 years, 25–34 years, 35–44 years, 45–54 years, 55–64 years, 65–74 years, 75–84 years, ≥85 years), calendar year, and nine US Census regional divisions (Pacific, Mountain, West North Central, West South Central, East North Central, East South Central, South Atlantic, Middle Atlantic, and New England).

**Table 2:** Tuberculosis treatment outcomes by race and ethnicity among US-born people diagnosed with tuberculosis, USA, 2003–19

	Treatment longer than 12 months*		Treatment discontinuation†		Death during treatment‡	
			
	cases/N (%)	aRR§ (95% CI)	cases/N (%)	aRR§ (95% CI)	cases/N (%)	aRR§ (95% CI)

White	1888/18 902 (10·0%)	Ref	506/22 492 (2·2%)	Ref	2489/22 492 (11·1%)	Ref
Black	2951/24 534 (12·0%)	1·20 (1·13–1·26)	862/28 789 (3·0%)	1·27 (1·13–1·42)	2869/28 789 (10·0%)	1·32 (1·25–1·38)
Hispanic	1179/11 138 (10·6%)	1·09 (1·01–1·17)	365/12 666 (2·9%)	1·19 (1·04–1·38)	725/12 666 (5·7%)	1·19 (1·10–1·29)
Asian	171/2026 (8·4%)	0·96 (0·82–1·12)	22/2225 (1·0%)	0·50 (0·31–0·75)	91/2225 (4·1%)	1·10 (0·90–1·33)
American Indian or Alaska Native	199/1752 (11·4%)	1·20 (1·04–1·37)	40/2081 (1·9%)	0·76 (0·54–1·03)	236/2081 (11·3%)	1·47 (1·29–1·66)
Native Hawaiian or other Pacific Islander	33/364 (9·1%)	1·10 (0·77–1·49)	8/401 (2·0%)	0·99 (0·45–1·85)	19/401 (4·7%)	1·30 (0·82–1·91)
Other ethnicities	17/244 (7·0%)	0·83 (0·50–1·27)	7/289 (2·4%)	1·11 (0·48–2·12)	22/289 (7·6%)	1·25 (0·83–1·77)

Results exclude non-US-born patients and patients diagnosed with multidrug-resistant tuberculosis.

* Based on length of therapy reported by patients. Excludes negative values (n=5), outliers (n=2), patients who stopped therapy before completion within 12 months (n=7860) because of death or treatment discontinuation, and patients who took longer than 12 months for clinical reasons (n=1235).

† Includes patients who refused treatment or were lost.

‡ Includes any deaths that occurred between initiation of treatment and treatment completion or discontinuation.

§ Adjusted relative risks adjusting for sex, categorical age (<1 year, 1–4 years, 5–14 years, 15–24 years, 25–34 years, 35–44 years, 45–54 years, 55–64 years, 65–74 years, 75–84 years, ≥85 years), calendar year, and nine US Census regional divisions (Pacific, Mountain, West North Central, West South Central, East North Central, East South Central, South Atlantic, Middle Atlantic, and New England).

## Data Availability

The National Tuberculosis Surveillance System operates under an Assurance of Confidentiality issued by the Centers for Disease Control and Prevention (CDC) under Sections 306 and 308(d) of the Public Health Service Act (42 USC 242k and 242m(d)). Data are reported voluntarily to CDC by state and local health departments on a case report form called the Report of Verified Case of Tuberculosis (Office of Management and Budget number 0920–0026). The Assurance of Confidentiality prevents disclosure of any information that could be used to directly or indirectly identify patients. For more information, please see the CDC and Agency for Toxic Substances and Disease Registry policy on releasing and sharing data (http://www.cdc.gov/maso/Policy/ReleasingData.pdf). A limited dataset is available at http://wonder.cdc.gov/tuberculosis-v2013.html. Researchers can apply to analyse additional data at the CDC headquarters by contacting TBInfo@cdc.gov.

## Research in context

### Evidence before this study

We searched PubMed from inception to July 11, 2023, to identify previous studies on this topic, using the keywords “United States” AND “tuberculosis” AND (“race” OR “disparities”) AND (“mortality” OR “diagnosis” OR “treatment”). This search returned 228 results, which we reviewed for relevance. Of these, all but 13 were deemed not relevant to the present study because of the following reasons: the study setting was outside the USA; the studies were on tuberculosis incidence, tuberculosis transmission, tuberculosis drug pricing, or a non- tuberculosis outcome; the study population consisted of patients from a single racial or ethnic group; or the paper was not a research study. Most studies on tuberculosis mortality were limited to a subgroup such as incarcerated people, people with HIV, pregnant women, or residents of a single state, and reported mixed results. One study that used national surveillance data during 1990–2006 reported higher risks of tuberculosis-related mortality for people who identified as a racial or ethnic minority, relative to non-Hispanic White people. Few studies looked at non-mortality outcomes along the continuum of care; a study restricted to incarcerated people found that Hispanic persons were less likely to complete tuberculosis treatment and a study using national surveillance data during 1993–2006 found that US-born Hispanic people were less likely to complete treatment in 12 months.

### Added value of this study

In this study we used US tuberculosis surveillance data from 2003–2019 to describe racial or ethnic disparities in tuberculosis diagnosis and treatment outcomes among US-born patients. We examined tuberculosis outcomes along the continuum of care, to assess progress towards reducing inequities and identify opportunities for targeted interventions. We observed disparities across racial and ethnic groups for several study outcomes, including risk of diagnosis after death, severity of case presentation, treatment discontinuation, and death during treatment. These disparities persisted over the study period.

### Implications of all the available evidence

Building on scarce previous evidence, our findings suggest that, although tuberculosis outcomes have improved over time among US-born patients, racially marginalised people still have a higher risk of poor tuberculosis outcomes along the continuum of care. Initiatives are needed to reduce time to diagnosis and improve treatment outcomes.
