# Supplementary material for: Racial and ethnic disparities in diagnosis and treatment outcomes among US-born people diagnosed with tuberculosis, 2003–19: an analysis of national surveillance data
Source: Lancet Public Health. Author manuscript; Available in PMC 2024 Dec 23. (PMC11665086; doi:10.1016/S2468-2667(23)00276-1)
Supplement: Appendix [file NIHMS2038324-supplement-Appendix.pdf]

THE LANCET
Public Health

Supplementary appendix

This appendix formed part of the original submission and has been peer reviewed. We post it as supplied by the authors.

Supplement to: Regan M, Li Y, Swartwood NA, et al. Racial and ethnic disparities in diagnosis and treatment outcomes among US-born people diagnosed with tuberculosis, 2003–19: an analysis of national surveillance data. *Lancet Public Health* 2023; **8:** e47–56.

SUPPLEMENTARY APPENDIX

Racial/ethnic disparities in case presentation and outcomes of care among US-born persons diagnosed with tuberculosis, 2003-2019

Table of Contents

Table S1: Demographic characteristics by race/ethnicity among US-born individuals diagnosed with tuberculosis, 2003-2019.. 1
Figure S1: Flow chart of outcomes..2
Figure S2: Total number of cases over time by race/ethnicity for case presentation and diagnosis outcomes.............3
Figure S3: Estimated risk of select TB outcomes over time relative to White persons, adjusted for sex, age category, geographic region, and year...4
Table S2: Sensitivity analysis – Diagnosis after death by race/ethnicity among US-born persons diagnosed with TB, United States 2003-2019 6
Table S3: Sensitivity analysis – cavitation by race/ethnicity among US-born persons diagnosed with TB, United States 2003-2019...7
Table S4: Sensitivity analysis – smear positivity by race/ethnicity among US-born persons diagnosed with TB, United States, 2003-2019.. 8
Table S5: Sensitivity analysis - treatment > 12 months by race/ethnicity among US-born persons diagnosed with TB, United States, 2003-2019.. 9
Table S6: Sensitivity analysis - treatment discontinuation by race ethnicity among US-born persons diagnosed with TB, United States 2003-2019..10
Table S7: Sensitivity analysis -death during treatment by race/ethnicity among US-born persons diagnosed with TB, United States, 2003-2019……………………..11
Figure S4: Estimated predicted probabilities of TB case presentation and treatment outcomes over time, adjusted for sex, age category, geographic region, and year…...12
Table S8: Time trends in TB treatment outcomes by race/ethnicity among US-born persons diagnosed with TB, United States, 2003-2019 …………………...15
Table S9: TB diagnosis outcomes by race/ethnicity among US-born persons diagnosed with TB including persons with MDR-TB, United States 2003-2018……………………...16
Table S10: TB treatment outcomes by race/ethnicity among US-born persons diagnosed with TB including persons with MDR-TB, United States 2003-2018……………………..17
Table S11: Disparities among US-born Hispanic persons by racial/ethnic subgroups 2003-2019.............................18

Table S1: Demographic characteristics by race/ethnicity among US-born individuals diagnosed with tuberculosis, 2003-2019

	Race/ethnicity							
	**White n (%)**	**Black n (%)**	**Hispanic n (%)**	**Asian n (%)**	**American Indian/Alaska Native n (%)**	**Native Hawaiian/other Pacific Islander n (%)**	**Other n (%)**	**Total n (%)**
**Total**	23,822 (32·9)	30,198 (41·7)	13,181 (18·2)	2,268 (3·1)	2,176 (3·0)	425 (0·6)	299 (0·4)	72,369
**Male**	16,235 (68·2)	19,453 (64·4)	7,875 (59·7)	1,242 (54·8)	1,305 (60·0)	233 (54·8)	93 (64·5)	46,536 (64·3)
**Age group (years)**								
**<1 year**	94 (0·4)	276 (0·9)	615 (4·7)	141 (6·2)	15 (0·7)	18 (4·2)	6 (2·0)	1,165 (1·6)
**1-4 years**	335 (1·4)	1,016 (3·4)	2,026 (15·4)	403 (17·8)	88 (4·0)	81 (19·1)	41 (13·7)	3,990 (5·5)
**5-14 years**	194 (0·8)	755 (2·5)	1,146 (8·7)	252 (11·1)	84 (3·9)	54 (12·7)	17 (5·7)	2,502 (3·5)
**15-24 years**	771 (3·2)	2,160 (7·2)	2,031 (15·4)	558 (24·6)	168 (7·7)	65 (15·3)	29 (9·7)	5,782 (8·0)
**25-34 years**	1,489 (6·3)	3,253 (10·8)	1,598 (12·1)	361 (15·9)	194 (8·9)	55 (12·9)	32 (10·7)	6,982 (9·6)
**35-44 years**	2,751 (11·5)	4,892 (16·2)	1,448 (11·0)	137 (6·0)	328 (15·1)	38 (8·9)	43 (14·4)	9,637 (13·3)
**45-54 years**	4,853 (20·4)	6,999 (23·2)	1,474 (11·2)	92 (4·1)	467 (21·5)	33 (7·8)	38 (12·7)	13,956 (19·3)
**55-64 years**	4,661 (19·6)	5,223 (17·3)	1,164 (8·8)	86 (3·8)	364 (16·7)	39 (9·2)	48 (16·1)	11,585 (16·0)
**65-74 years**	3,305 (13·9)	2,765 (9·2)	837 (6·4)	72 (3·2)	232 (10·7)	23 (5·4)	22 (7·4)	7,256 (10·0)
**75-84 years**	3,523 (14·8)	1,952 (6·5)	609 (4·6)	94 (4·1)	164 (7·5)	17 (4·0)	13 (4·3)	6,372 (8·8)
**85+ years**	1,844 (7·7)	906 (3·0)	233 (1·8)	72 (3·2)	72 (3·3)	2 (0·5)	10 (3·3)	3,139 (4·3)
**Regional division**								
**New England**	664 (2·8)	349 (1·2·0)	243 (1·8)	65 (2·9)	3 (0·1)	1 (0·2)	2 (0·7)	1,327 (1·8)
**Middle Atlantic**	2,216 (9·3)	3,396 (11·2)	1,446 (11·0)	247 (10·9)	5 (0·2)	6 (1·4)	28 (9·4)	7,344 (10·1)
**East North Central**	2,715 (11·4)	3,604 (11·9)	649 (4·9)	196 (8·6)	36 (1·7)	9 (2·1)	8 (2·7)	7,217 (10·0)
**West North Central**	1,065 (4·5)	615 (2·0)	204 (1·5)	72 (3·2)	209 (9·6)	38 (8·9)	13 (4·3)	2,216 (3·1)
**South Atlantic**	5,554 (23·3)	10,749 (35·6)	1,299 (9·9)	276 (12·2)	165 (7·6)	16 (3·8)	68 (22·7)	18,127 (25·0)
**East South Central**	2,916 (12·2)	3,409 (11·3)	183 (1·4)	27 (1·2)	18 (0·8)	6 (1·4)	8 (2·7)	6,567 (9·1)
**West South Central**	4,541 (19·1)	5,753 (19·1)	4,373 (33·2)	230 (10·1)	307 (14·1)	95 (22·4)	86 (28·8)	15,385 (21·3)
**Mountain**	1,030 (4·3)	284 (0·9)	960 (7·3)	71 (3·1)	545 (25·0)	16 (3·8)	13 (4·3)	2,919 (4·0)
**Pacific**	3,121 (13·1)	2,039 (6·8)	3,824 (29·0)	1,084 (47·8)	888 (40·8)	238 (56·0)	73 (24·4)	11,267 (15·6)
**Disease site***								
**Pulmonary**	18,940 (79·5)	21,783 (72·2)	9,135 (69·3)	1,583 (69·9)	1,620 (74·5)	260 (61·3)	217 (72·6)	53,538 (74·0)
**Extrapulmonary**	3,329 (14·0)	5,491 (18·2)	2,681 (20·3)	430 (19·0)	336 (15·4)	85 (20·0)	50 (16·7)	12,402 (17·1)
**Both**	1,544 (6·5)	2,907 (9·6)	1,364 (10·3)	253 (11·2)	219 (10·1)	79 (18·6)	32 (10·7)	6,398 (8·8)

*Disease site unknown for 31 persons

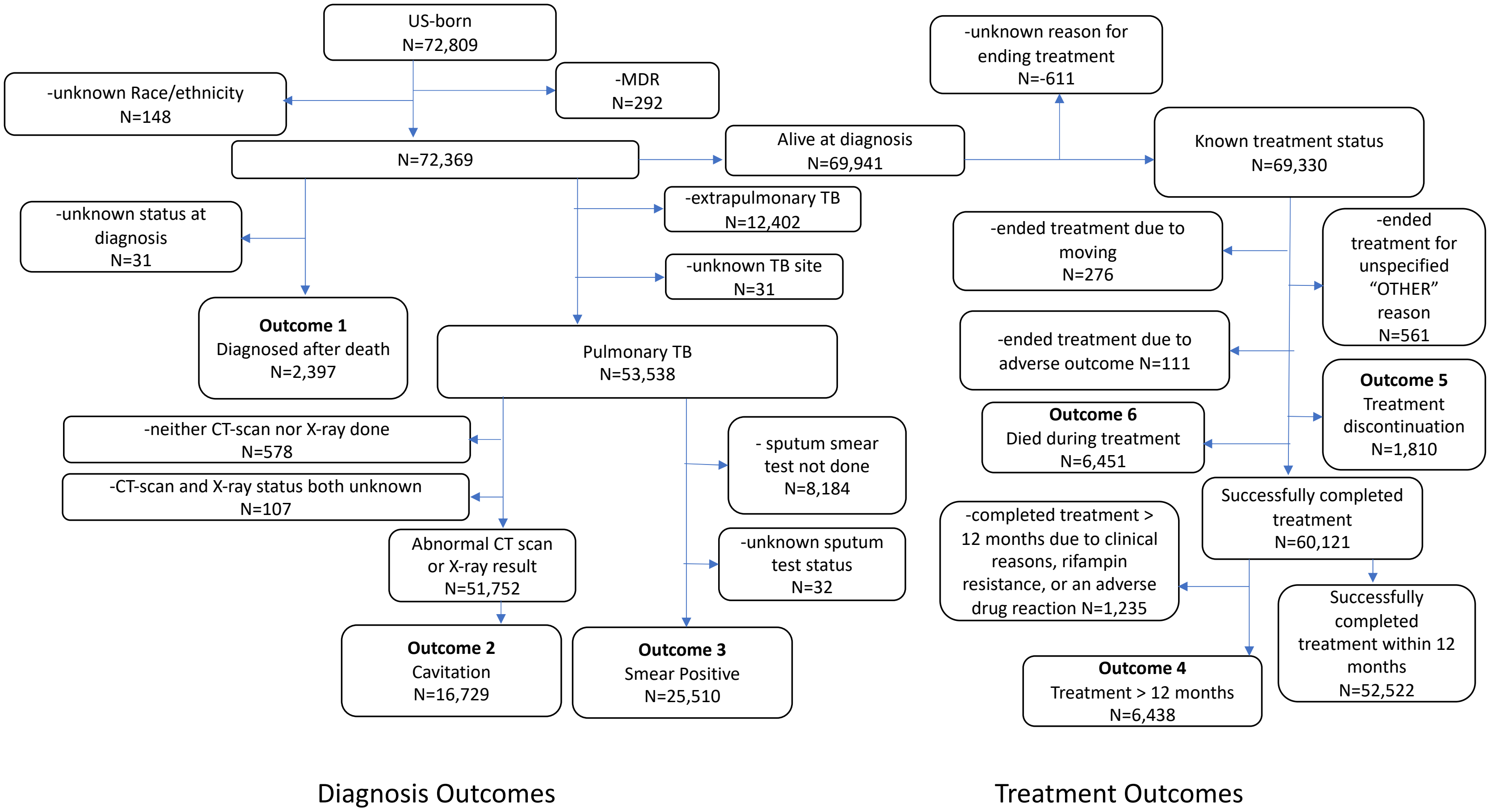

Figure S1: Flow chart of outcomes

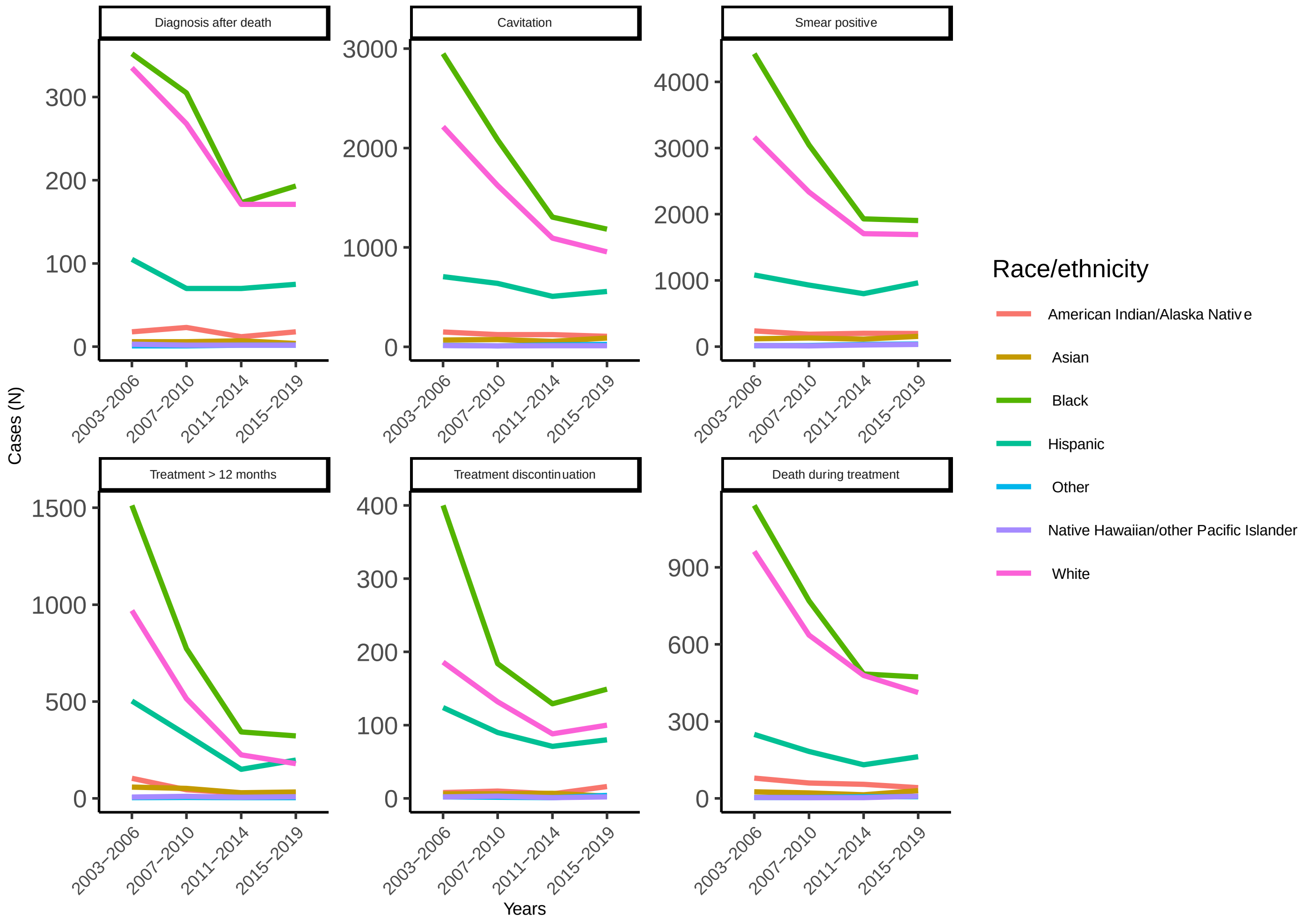

Figure S2: **Total number of cases over time by race/ethnicity for case presentation and diagnosis outcomes**

Diagnosis after death

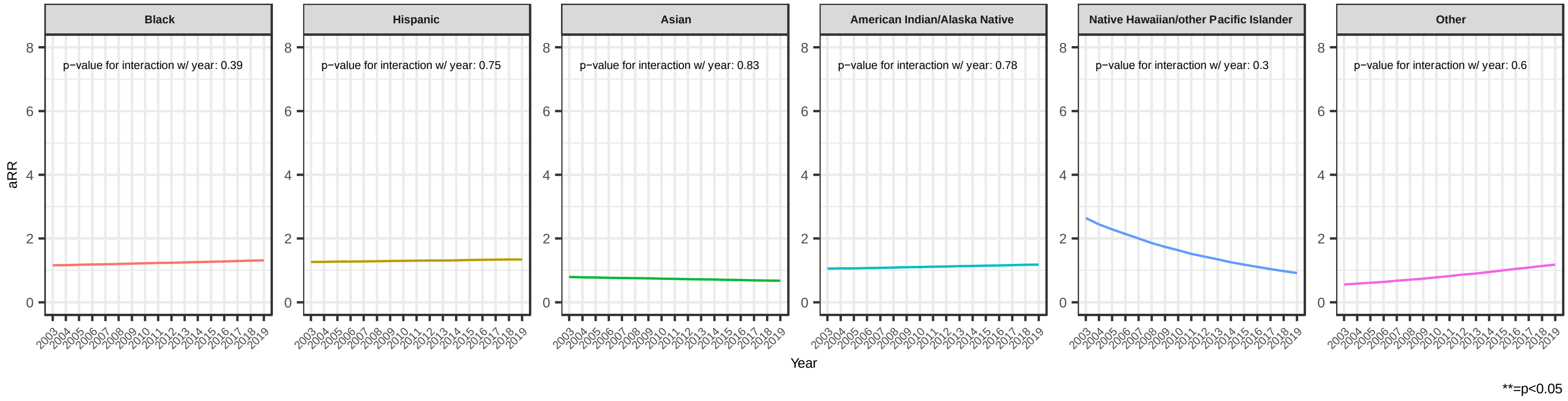

Cavitation

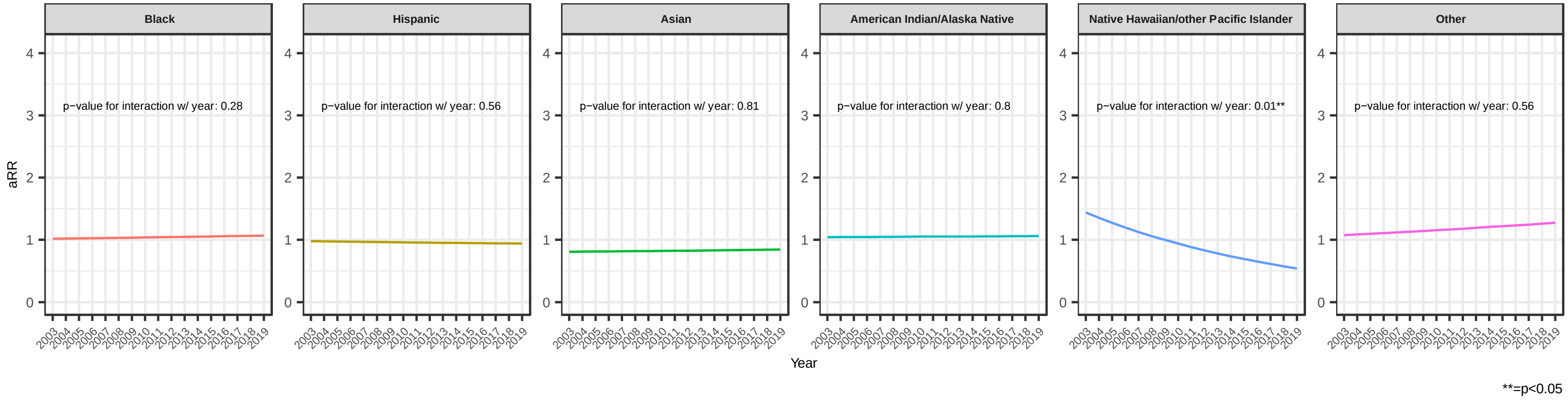

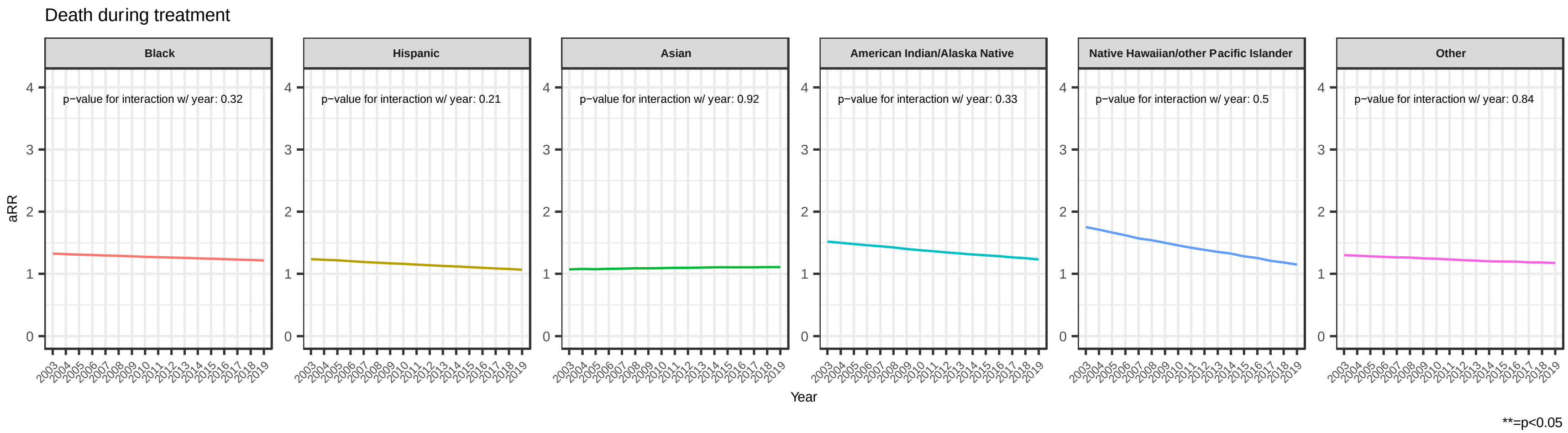

Figure S3: Estimated index of disparity over time for case presentation and treatment outcomes. Solid line indicates point estimates and shaded region indicates 95% confidence intervals. Cavitation estimates are restricted to persons with pulmonary TB and a recorded abnormal radiograph or computed tomography (CT) scan result.

Table S2: Sensitivity analysis – Diagnosis after death by race/ethnicity among US-born persons diagnosed with TB, United States 2003-2019

	Adjusting for age and sex only aRR (95% CI)	Adjusting for division (*Table 1 model*) aRR* (95% CI)	Adjusting for region aRR* (95% CI)	Adjusting for urban/rural aRR* (95% CI)
**White**	Ref	Ref	Ref	Ref
**Black**	1·23 (1·13–1·34)	1·22 (1·12–1·33)	1·21 (1·11–1·33)	1·26 (1·15–1·37)
**Hispanic**	1·32 (1·16–1·50)	1·30 (1·14–1·47)	1·33 (1·17–1·51)	1·35 (1·18–1·53)
**Asian**	0·67 (0·43–0·98)	0·74 (0·48–1·09)	0·71 (0·45–1·04)	0·68 (0·44–1·00)
**American Indian/Alaska Native**	1·15 (0·90–1·44)	1·09 (0·85–1·39)	1·22 (0·95–1·54)	1·12 (0·87–1·41)
**Native Hawaiian/other Pacific Islander**	1·40 (0·68–2·49)	1·54 (0·75–2·75)	1·49 (0·72–2·65)	1·40 (0·68–2·49)
**Other**	0·88 (0·35–1·75)	0·88 (0·35–1·75)	0·88 (0·35–1·74)	0·87 (0·35–1·74)
**Akaike information criterion (AIC)**	20940·31	19367·3	19377·4	19378·9

	Adjusting for categorical time (4 periods) aRR** (95% CI)	Adjusting for year dummy variables aRR** (95% CI)	Model with a cubic spline for time aRR** (95% CI)	Adjusting for time random effects for time aRR** (95% CI)
**White**	Ref	Ref	Ref	Ref
**Black**	1·22 (1·11–1·33)	1·22 (1·12–1·33)	1·22 (1·12–1·33)	1·22 (1·12–1·33)
**Hispanic**	1·29 (1·13–1·47)	1·30 (1·14–1·47)	1·30 (1·14–1·47)	1·30 (1·14–1·48)
**Asian**	0·75 (0·47–1·11)	0·74 (0·47–1·09)	0·74 (0·48–1·09)	0·74 (0·49–1·12)
**American Indian/Alaska Native**	1·14 (0·88–1·45)	1·09 (0·85–1·39)	1·09 (0·85–1·39)	1·09 (0·85–1·40)
**Native Hawaiian/other Pacific Islander**	1·62 (0·79–2·88)	1·56 (0·76–2·78)	1·54 (0·75–2·75)	1·55 (0·81–2·97)
**Other**	0·80 (0·29–1·70)	0·90 (0·36–1·79)	0·88 (0·35–1·75)	0·89 (0·40–1·96)
**Akaike information criterion (AIC)**	18701·5	19377·4	19370·5	19367·4

*Also adjusted for categorical age, sex, and calendar year
** Also adjusted for sex, categorical age, and regional division

Table S3: Sensitivity analysis – cavitation by race/ethnicity among US-born persons diagnosed with TB, United States 2003-2019

	All TB cases (pulmonary and/or extrapulmonary) aRR* (95% CI)	Pulmonary TB cases + concomitant pulmonary and extrapulmonary aRR* (95% CI)	Pulmonary only TB cases excluding HIV positive [a,b] aRR* (95% CI)	Pulmonary only TB cases,confirmed HIV negative [a,b] aRR* (95% CI)	Adjusting for age and sex only aRR (95% CI)	Adjusting for division [a] (*Table 1 model*) aRR* (95% CI)
**White**	Ref	Ref	Ref	Ref	Ref	Ref
**Black**	0·94 (0·91–0·96)	0·99 (0·97–1·02)	1·10 (1·05–1·15)	1·05 (1·00–1·11)	1·05 (1·02–1·08)	1·08 (1·06–1·10)
**Hispanic**	0·88 (0·84–0·91)	0·92 (0·88–1·06)	0·99 (0·92–1·05)	0·93 (0·87–1·00)	0·96 (0·92–1·00)	1·08 (1·05–1·10)
**Asian**	0·74 (0·67–0·82)	0·79 (0·71–0·87)	0·83 (0·71–0·96)	0·77 (0·66–0·90)	0·73 (0·65–0·80)	0·95 (0·89–1·01)
**American Indian/Alaska Native**	1·01 (0·93–1·08)	1·02 (0·94–1·10)	1·09 (0·96–1·22)	1·06 (0·94–1·19)	0·96 (0·92–1·00)	1·06 (1·01–1·11)
**Native Hawaiian/other Pacific Islander**	0·77 (0·60–0·96)	0·82 (0·64–1·01)	1·24 (0·97–1·51)	0·77 (0·55–1·04)	0·79 (0·61–0·99)	1·08 (0·93–1·22)
**Other**	1·11 (0·91–1·32)	0·82 (0·64–1·01)	0·83 (0·57–1·14)	1·21 (0·95–1·48)	1·13 (0·93–1·33)	1·12 (0·98–1·25)
**Akaike information criterion (AIC)**	73761·4	21223·6	19057·1	20849·13	61378·09	60671·7

	Adjusting for region [a] aRR* (95% CI)	Adjusting for urban/rural [a] aRR* (95% CI)	Adjusting for categorical time (4 periods) [a] aRR** (95% CI)	Adjusting for year dummy variables [a] aRR** (95% CI)	Model with a cubic spline for time [a] aRR** (95% CI)	Adjusting for time random effects for time [a] aRR** (95% CI)
**White**	Ref	Ref	Ref	Ref	Ref	Ref
**Black**	1·02 (1·00–1·05)	1·08 (1·05–1·11)	1·08 (1·06–1·10)	1·08 (1·06–1·10)	1·08 (1·06–1·10)	1·08 (1·06–1·10)
**Hispanic**	1·01 (0·97–1·05)	0·99 (0·95–1·03)	1·08 (1·05–1·11)	1·07 (1·05–1·10)	1·07 (1·05–1·10)	1·08 (1·05–1·10)
**Asian**	0·81 (0·73–0·90)	0·77 (0·70–0·86)	0·95 (0·89–1·02)	0·95 (0·89–1·01)	0·95 (0·89–1·01)	0·95 (0·89–1·01)
**American Indian/Alaska Native**	1·07 (0·99–1·15)	0·90 (0·84–0·97)	1·06 (1·01–1·11)	1·06 (1·01–1·11)	1·06 (1·01–1·11)	1·06 (1·01–1·11)
**Native Hawaiian/other Pacific Islander**	0·87 (0·67–1·09)	0·80 (0·61–1·00)	1·05 (0·90–1·20)	1·08 (0·93–1·22)	1·08 (0·93–1·22)	1·08 (0·94–1·23)
**Other**	1·18 (0·98–1·39)	1·11 (0·91–1·31)	1·10 (0·96–1·24)	1·12 (0·98–1·25)	1·12 (0·98–1·25)	1·12 (0·99–1·26)
**Akaike information criterion (AIC)**	61016·1	61130·5	58639·1	60656·7	60646·5	60661·3

[*] Also adjusted for categorical age, sex, and calendar year
[**] Also adjusted for sex, categorical age, and regional division
[a.] Among pulmonary cases with abnormal X-ray or CT scan
[b.] From 2011 onwards as HIV data not reported for CA before this year.

Table S4: Sensitivity analysis – smear positivity by race/ethnicity among US-born persons diagnosed with TB, United States, 2003-2019

	All TB cases (pulmonary and/or extrapulmonary) aRR* (95% CI)	Pulmonary TB cases + concomitant pulmonary and extrapulmonary aRR* (95% CI)	Pulmonary only TB excluding HIV positive [a,b] aRR* (95% CI)	Pulmonary onlyTB cases, confirmed HIV negative [a,b] aRR* (95% CI)	Adjusting for age and sex only aRR (95% CI)	Adjusting for division [a] (*Table 1 model*) aRR* (95% CI)
**White**	Ref	Ref	Ref	Ref	Ref	Ref
**Black**	1·00 (0·98–1·02)	1·05 (1·03–1·07)	1·07 (1·04–1·11)	1·08 (1·04–1·11)	1·08 (1·06–1·10)	1·08 (1·06–1·10)
**Hispanic**	1·02 (0·99–1·05)	1·05 (1·02–1·08)	1·06 (1·02–1·10)	1·06 (1·02–1·11)	1·07 (1·04–1·10)	1·08 (1·05–1·10)
**Asian**	0·89 (0·83–0·95)	0·92 (0·86–0·98)	0·93 (0·85–1·02)	0·94 (0·86–1·03)	0·94 (0·88–1·00)	0·95 (0·89–1·01)
**American Indian/Alaska Native**	1·08 (1·02–1·13)	1·06 (1·01–1·11)	1·06 (0·98–1·14)	1·05 (0·97–1·13)	1·05 (1·01–1·10)	1·06 (1·01–1·11)
**Native Hawaiian/other Pacific Islander**	0·93 (0·80–1·06)	1·01 (0·88–1·14)	1·18 (1·00–1·35)	1·19 (1·00–1·37)	1·08 (0·93–1·22)	1·08 (0·93–1·22)
**Other**	1·10 (0·96–1·23)	1·12 (0·99–1·25)	1·13 (0·96–1·29)	1·09 (0·91–1·25)	1·10 (0·97–1·23)	1·12 (0·98–1·25)
**Akaike information criterion (AIC)**	76506·4	60710·32	21473·3	19414·3	67893·57	60671·7

	Adjusting for region [a] aRR* (95% CI)	Adjusting for urban/rural [a] aRR* (95% CI)	Adjusting for categorical time (4 periods) [a] aRR** (95% CI)	Adjusting for year dummy variables [a] aRR** (95% CI)	Model with a cubic spline for time [a] aRR** (95% CI)	Adjusting for time random effects for time [a] aRR** (95% CI)
**White**	Ref	Ref	Ref	Ref	Ref	Ref
**Black**	1·08 (1·06–1·10)	1·09 (1·07–1·11)	1·08 (1·06–1·10)	1·08 (1·06–1·10)	1·08 (1·06–1·10)	1·08 (1·06–1·10)
**Hispanic**	1·08 (1·05–1·11)	1·08 (1·06–1·11)	1·08 (1·05–1·11)	1·07 (1·05–1·10)	1·07 (1·05–1·10)	1·08 (1·05–1·10)
**Asian**	0·95 (0·89–1·01)	0·96 (0·90–1·02)	0·95 (0·89–1·02)	0·95 (0·89–1·01)	0·95 (0·89–1·01)	0·95 (0·89–1·01)
**American Indian/Alaska Native**	1·06 (1·01–1·11)	1·03 (0·98–1·08)	1·06 (1·01–1·11)	1·06 (1·01–1·11)	1·06 (1·01–1·11)	1·06 (1·01–1·11)
**Native Hawaiian/other Pacific Islander**	1·07 (0·93–1·21)	1·07 (0·92–1·21)	1·05 (0·90–1·20)	1·08 (0·93–1·22)	1·08 (0·93–1·22)	1·08 (0·94–1·23)
**Other**	1·11 (0·98–1·24)	1·09 (0·95–1·22)	1·10 (0·96–1·24)	1·12 (0·98–1·25)	1·12 (0·98–1·25)	1·12 (0·99–1·26)
**Akaike information criterion (AIC)**	60683·2	60599·6	58639·1	60656·7	60646·5	60661·3

*Also adjusted for categorical age, sex, and calendar year

** Also adjusted for sex, categorical age, and regional division

[a.] Among pulmonary cases with abnormal X-ray or CT scan

[b.] From 2011 onwards as HIV data not reported for CA before this year.

Table S5: Sensitivity analysis - treatment > 12 months by race/ethnicity among US-born persons diagnosed with TB, United States, 2003-2019

	Adjusting for age and sex only aRR(95% CI)	Adjusting for division (*Table 1 model*) aRR* (95% CI)	Adjusting for region aRR* (95% CI)	Adjusting for urban/rural aRR* (95% CI)
**White**	Ref	Ref	Ref	Ref
**Black**	1·18 (1·11–1·24)	1·20 (1·13–1·26)	1·16 (1·10–1·23)	1·13 (1·07–1·20)
**Hispanic**	1·12 (1·04–1·21)	1·09 (1·01–1·17)	1·15 (1·07–1·24)	1·16 (1·07–1·25)
**Asian**	0·94 (0·80–1·09)	0·97 (0·82–1·12)	0·99 (0·85–1·15)	1·01 (0·86–1·18)
**American Indian/Alaska Native**	1·14 (0·99–1·30)	1·20 (1·04–1·37)	1·20 (1·04–1·37)	1·26 (1·09–1·44)
**Native Hawaiian/other Pacific Islander**	0·98 (0·69–1·34)	1·10 (0·77–1·49)	1·16 (0·82–1·57)	1·20 (0·84–1·62)
**Other**	0·72 (0·44–1·11)	0·83 (0·50–1·27)	0·85 (0·51–1·29)	0·86 (0·52–1·30)
**Akaike information criterion AIC)**	40367·93	39500·6	39645·4	39672·9

	Adjusting for categorical time (4 periods) aRR** (95% CI)	Adjusting for year dummy variables aRR** (95% CI)	Model with a cubic spline for time aRR** (95% CI)	Adjusting for time random effects for time aRR** (95% CI)
**White**	Ref	Ref	Ref	Ref
**Black**	1·19 (1·13–1·26)	1·19 (1·13–1·26)	1·19 (1·13–1·26)	1·19 (1·13–1·26)
**Hispanic**	1·09 (1·01–1·17)	1·09 (1·01–1·18)	1·09 (1·01–1·18)	1·09 (1·01–1·18)
**Asian**	0·95 (0·81–1·11)	0·97 (0·83–1·13)	0·97 (0·83–1·13)	0·97 (0·83–1·13)
**American Indian/Alaska Native**	1·20 (1·04–1·38)	1·20 (1·04–1·37)	1·20 (1·04–1·38)	1·19 (1·04–1·37)
**Native Hawaiian/other Pacific Islander**	1·09 (0·76–1·49)	1·10 (0·77–1·49)	1·09 (0·77–1·49)	1·09 (0·79–1·52)
**Other**	0·81 (0·48–1·25)	0·84 (0·51–1·28)	0·84 (0·51–1·27)	0·84 (0·53–1·32)
**Akaike information criterion (AIC)**	38478·8	39403·0	39412·6	39451·3

*Also adjusted for categorical age, sex, and calendar year

** Also adjusted for sex, categorical age, and regional division

Table S6: Sensitivity analysis - treatment discontinuation by race ethnicity among US-born persons diagnosed with TB, United States 2003-2019

	Adjusting for age and sex only<br>aRR(95% CI)	Adjusting for division (*Table 1 model*)<br>aRR* (95% CI)	Adjusting for region<br>aRR* (95% CI)	Adjusting for urban/rural<br>aRR* (95% CI)
**White**	Ref	Ref	Ref	Ref
**Black**	1·07 (0·97–1·19)	1·27 (1·13–1·42)	1·21 (1·08–1·35)	1·11 (1·00–1·24)
**Hispanic**	1·27 (1·12–1·43)	1·19 (1·04–1·38)	1·43 (1·24–1·65)	1·29 (1·12–1·49)
**Asian**	0·63 (0·44–0·86)	0·50 (0·31–0·75)	0·51 (0·32–0·77)	0·47 (0·30–0·70)
**American Indian/Alaska Native**	0·74 (0·54–0·97)	0·76 (0·54–1·03)	0·82 (0·59–1·12)	0·92 (0·66–1·26)
**Native Hawaiian/other Pacific Islander**	1·32 (0·75–2·12)	0·99 (0·45–1·85)	1·05 (0·48–1·95)	1·06 (0·49–1·97)
**Other**	1·08 (0·52–1·93)	1·11 (0·48–2·12)	1·18 (0·51–2·26)	1·16 (0·50–2·23)
**Akaike information criterion (AIC)**	19140·03	15979·2	16197·7	16158·1

	Adjusting for categorical time (4 periods)<br>aRR** (95% CI)	Adjusting for year dummy variables<br>aRR** (95% CI)	Model with a cubic spline for time<br>aRR** (95% CI)	Adjusting for time random effects for time<br>aRR** (95% CI)
**White**	Ref	Ref	Ref	Ref
**Black**	1·24 (1·11–1·39)	1·27 (1·14–1·42)	1·27 (1·13–1·42)	1·27 (1·14–1·42)
**Hispanic**	1·20 (1·03–1·38)	1·19 (1·03–1·37)	1·19 (1·03–1·37)	1·19 (1·03–1·37)
**Asian**	0·52 (0·33–0·79)	0·49 (0·31–0·74)	0·49 (0·31–0·74)	0·49 (0·32–0·76)
**American Indian/Alaska Native**	0·70 (0·49–0·97)	0·76 (0·54–1·03)	0·76 (0·54–1·03)	0·75 (0·55–1·04)
**Native Hawaiian/other Pacific Islander**	1·04 (0·47–1·93)	0·99 (0·45–1·84)	0·98 (0·45–1·82)	0·99 (0·49–1·97)
**Other**	1·01 (0·41–2·03)	1·10 (0·47–2·10)	1·10 (0·48–2·11)	1·10 (0·53–2·28)
**Akaike information criterion (AIC)**	15350·5	15969·5	15960·5	15971·2

*Also adjusted for categorical age, sex, and calendar year

** Also adjusted for sex, categorical age, and regional division

Table S7: Sensitivity analysis -death during treatment by race/ethnicity among US-born persons diagnosed with TB, United States, 2003-2019

	Adjusting for age and sex only aRR(95% CI)	Adjusting for division (*Table 1 model*) aRR* (95% CI)	Adjusting for region aRR* (95% CI)	Adjusting for urban/rural aRR* (95% CI)
**White**	Ref	Ref	Ref	Ref
**Black**	1·28 (1·22–1·34	1·32 (1·25–1·38)	1·32 (1·26–1·39)	1·27 (1·20–1·33)
**Hispanic**	1·20 (1·11–1·29)	1·19 (1·10–1·29)	1·21 (1·12–1·31)	1·16 (1·07–1·25)
**Asian**	1·11 (0·92–1·31)	1·10 (0·90–1·33)	1·11 (0·91–1·33)	1·05 (0·86–1·26)
**American Indian/Alaska Native**	1·39 (1·23–1·55)	1·47 (1·29–1·66)	1·47 (1·30–1·66)	1·57 (1·39–1·77)
**Native Hawaiian/other Pacific Islander**	1·41 (0·95–1·97)	1·30 (0·82–1·91)	1·30 (0·82–1·91)	1·26 (0·79–1·84)
**Other**	1·22 (0·95–1·97)	1·25 (0·83–1·77)	1·26 (0·83–1·78)	1·26 (0·83–1·76)
**Akaike information criterion (AIC)**	39560·14	37212·2	37212·2	37140·3

	Adjusting for categorical time (4 periods) aRR** (95% CI)	Adjusting for year dummy variables [a] aRR** (95% CI)	Model with a cubic spline for time aRR** (95% CI)	Adjusting for time random effects for time aRR** (95% CI)
**White**	Ref	Ref	Ref	Ref
**Black**	1·32 (1·25–1·38)	1·32 (1·25–1·38)	1·32 (1·25–1·38)	1·32 (1·25–1·38)
**Hispanic**	1·19 (1·10–1·29)	1·19 (1·10–1·29)	1·19 (1·10–1·29)	1·19 (1·10–1·28)
**Asian**	1·13 (0·92–1·36)	1·10 (0·90–1·33)	1·10 (0·90–1·33)	1·10 (0·91–1·33)
**American Indian/Alaska Native**	1·45 (1·27–1·64)	1·47 (1·29–1·66)	1·47 (1·29–1·66)	1·46 (1·29–1·66)
**Native Hawaiian/other Pacific Islander**	1·23 (0·75–1·84)	1·31 (0·82–1·92)	1·30 (0·82–1·91)	1·29 (0·85–1·97)
**Other**	1·30 (0·85–1·84)	1·25 (0·83–1·77)	1·25 (0·83–1·77)	1·24 (0·85–1·81)
**Akaike information criterion (AIC)**	35925·8	37220·4	37210·9	37222·0

*Also adjusted for categorical age, sex, and calendar year
**Also adjusted for sex, categorical age, and regional division

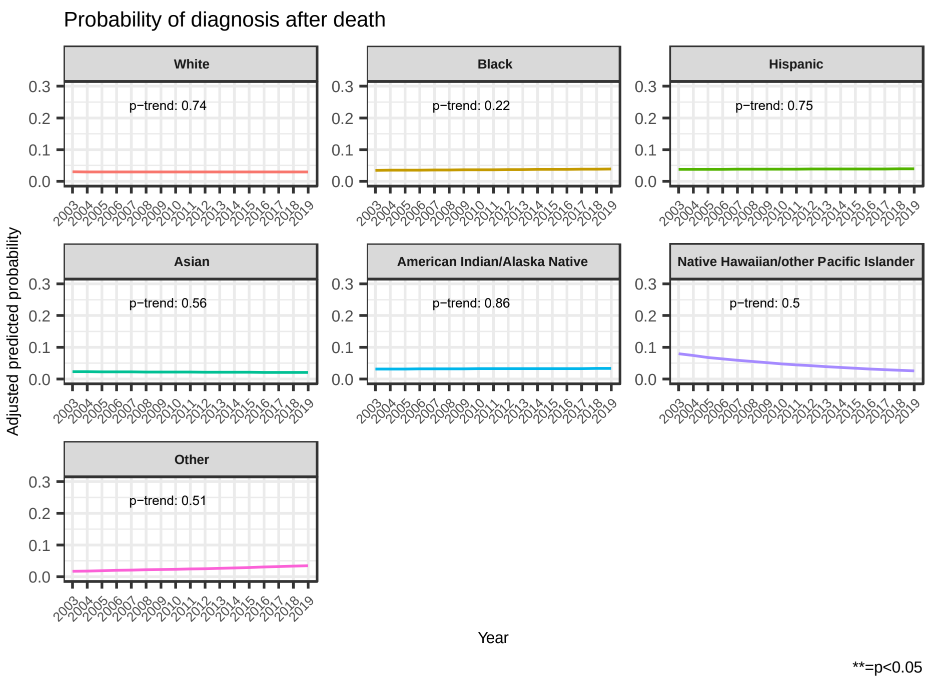
Probability of diagnosis after death
White
p−trend: 0.74
Black
p−trend: 0.22
Hispanic
p−trend: 0.75
Asian
p−trend: 0.56
American Indian/Alaska Native
p−trend: 0.86
Native Hawaiian/other Pacific Islander
p−trend: 0.5
Other
p−trend: 0.51
Adjusted predicted probability
Year
**=p<0.05

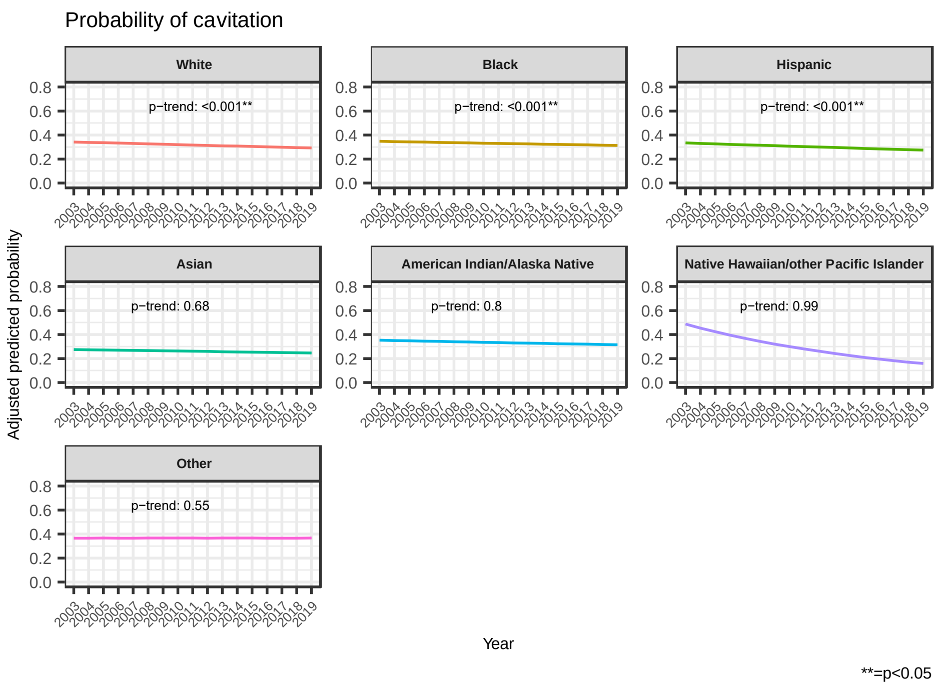
Probability of cavitation
White
p−trend: <0.001**
Black
p−trend: <0.001**
Hispanic
p−trend: <0.001**
Asian
p−trend: 0.68
American Indian/Alaska Native
p−trend: 0.8
Native Hawaiian/other Pacific Islander
p−trend: 0.99
Other
p−trend: 0.55
Adjusted predicted probability
Year
**=p<0.05

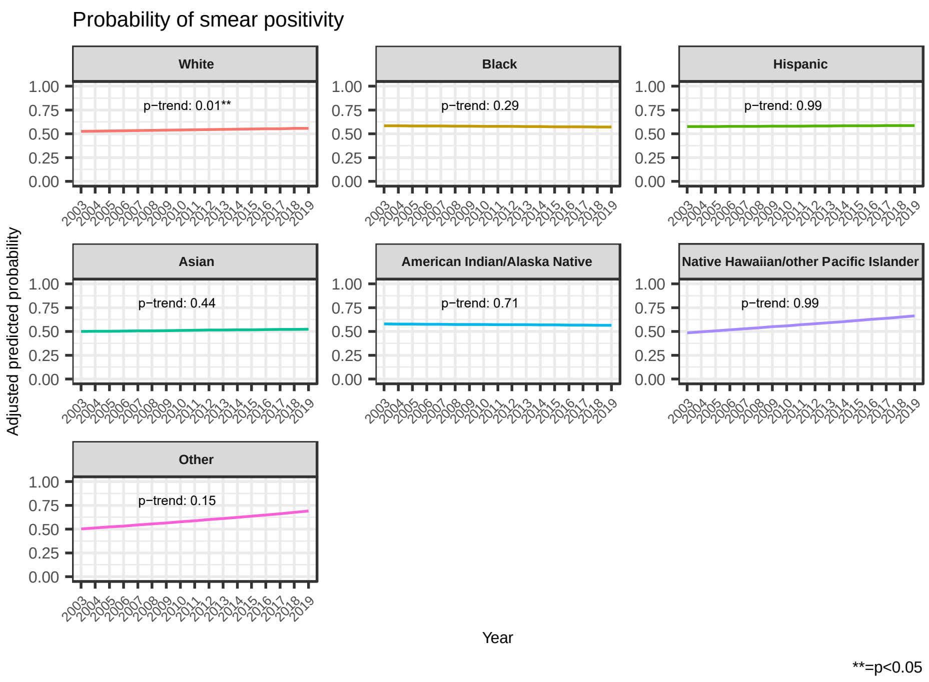
Probability of smear positivity
White
p−trend: 0.01**
Black
p−trend: 0.29
Hispanic
p−trend: 0.99
Asian
p−trend: 0.44
American Indian/Alaska Native
p−trend: 0.71
Native Hawaiian/other Pacific Islander
p−trend: 0.99
Other
p−trend: 0.15
Adjusted predicted probability
Year
**=p<0.05

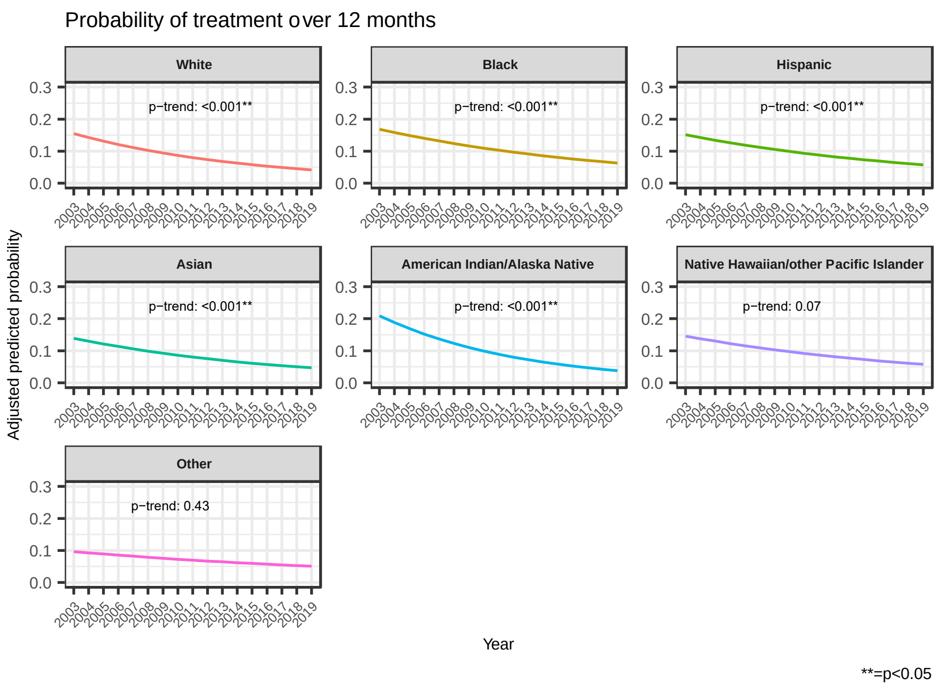
Probability of treatment over 12 months
White
p−trend: <0.001**
Black
p−trend: <0.001**
Hispanic
p−trend: <0.001**
Asian
p−trend: <0.001**
American Indian/Alaska Native
p−trend: <0.001**
Native Hawaiian/other Pacific Islander
p−trend: 0.07
Other
p−trend: 0.43
Adjusted predicted probability
Year
**=p<0.05

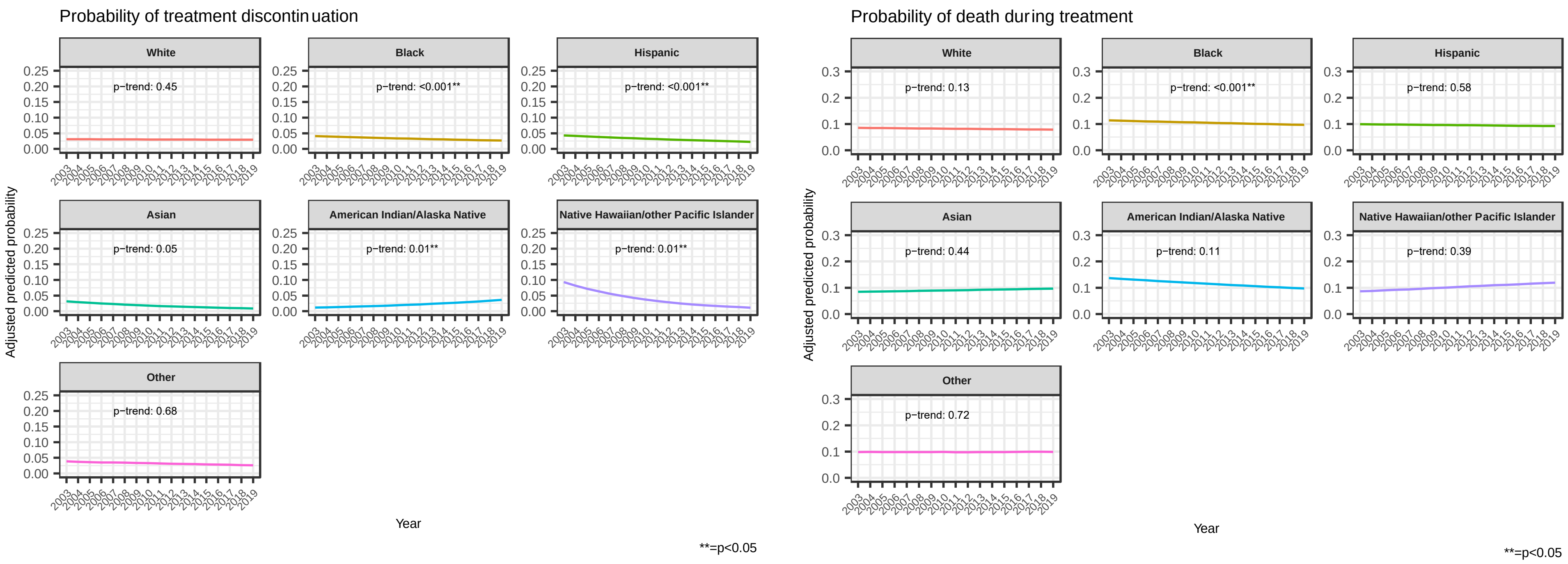

Figure S4: Estimated predicted probabilities of TB case presentation and treatment outcomes over time, adjusted for sex, age category, geographic region, and year. Cavitation estimates are restricted to persons with pulmonary TB and a recorded abnormal radiograph or computed tomography (CT) scan result. Sputum smear positive esimates are restricted to individials with pulmonary TB and a recorded AFB sputum smear result.

Table S8: Time trends* in TB treatment outcomes by race/ethnicity among US-born persons diagnosed with TB, United States, 2003-2019*

	Diagnosis after death Percent change (95% CI)	Cavitation [a] Percent change (95% CI)	Sputum smear positive [a] Percent change (95% CI)	Treatment > 12 months Percent change (95% CI)	Treatment discontinuation Percent change (95% CI)	Death during treatment Percent change (95% CI)
**White**	Ref	Ref	Ref	Ref	Ref	Ref
**Black**	13·7% (-13·4–45·9)	5·0% (-3·0–14·2)	-7·3% (-12·4– -1·8)	37·9% (12·9–65·7)	-29·4 % (-49·3– -4·6)	-7·5% (-20·3–8·0)
**Hispanic**	6·5% (-27·8–57·5)	-3·3% (-13·9–10·0)	-3·5% (-10·9–3·8)	39·2% (10·0–75·7)	-43·0% (-61·0– -19·6)	-13·1% (-31·7–9·2)
**Asian**	-16·9% (-76·1–215·0)	4·10% (-22·6–42·5)	-1·1% (-18·1–18·6)	25·5% (-20·1–107·4)	-68·8% (-89·5– -9·4)	2·6% (-36·6–80·4)
**American Indian/Alaska Native**	11·3% (-49·5–133·4)	2·1% (-18·4–26·3)	-6·9% (-19·7–7·0)	-30·9% (-56·7–12·8)	223·9% (18·3–835·3)	-16·8% (-41·8–19·8)
**Native Hawaiian/other Pacific Islander**	-64·1% (-95·0–158·9)	-58·0% (-80·1– -11·9)	27·2% (-18·5–95·2)	38·0% (-47·7–290·4)	-86·1% (-97·2– -30·7)	-29·6% (-77·7–95·0)
**Other**	88·1% (-85·7–2,050·3)	19·3% (-32·0–121·7)	27·2% (-14·4–90·9)	89·3% (-58·5–650·7)	-33·0% (-89·7–381·3)	-10·4% (-72·9–161·4)

*Percent change in adjusted risk over time relative to White persons, adjusted for sex, age category, regional division, and year. We calculated the percent change as follows: $((r_{2018}-r_{2003})/r_{2003})*100$.

[a] Cavitation estimates are restricted to persons with pulmonary TB and a recorded abnormal radiograph or computed tomography (CT) scan result. Sputum smear positive estimates are restricted to individuals with pulmonary TB and a recorded AFB sputum smear result.

Supplementary Table 9: TB diagnosis outcomes by race/ethnicity among US-born persons diagnosed with TB including persons with MDR-TB, United States 2003-2018*

Race/ethnicity	Diagnosis after death [a]	aRR** (95% CI)	Cavitation [b]	aRR**(95% CI)	Smear positive [c]	aRR**(95% CI)
**White**	916/23,144 (4·0%)	Ref	5,764/17,666 (32·6%)	Ref	8,645/15,988 (54·1%)	Ref
**Black**	987/29,372 (3·4%)	1·21 (1·11–1·33)	7,339/20,491 (35·8%)	1·03 (1·01–1·06)	11,002/18,705 (58·8%)	1·08 (1·06–1·10)
**Hispanic**	305/12,631 (2·4%)	1·28 (1·12–1·46)	2,321/8,488 (27·3%)	0·96 (0·92–1·00)	3,616/6,482 (55·8%)	1·08 (1·05–1·11)
**Asian**	22/2,191 (1·0%)	0·74 (0·47–1·10)	288/1,494 (19·3%)	0·83 (0·75–0·92)	511/1,071 (47·7%)	0·96 (0·90–1·02)
**American Indian/Alaska Native**	71/2,098 (3·4%)	1·14 (0·88–1·45)	492/1,525 (32·3%)	1·05 (0·97–1·13)	797/1,416 (56·3%)	1·06 (1·01–1·11)
**Native Hawaiian/other Pacific Islander**	9/400 (2·3%)	1·61 (0·78–2·86)	47/229 (20·5%)	0·92 (0·71–1·15)	81/151 (53·6%)	1·06 (0·91–1·20)
**Other**	5/282 (1·8%)	0·79 (0·29–1·67)	67/195 (34·4%)	1·15 (0·95–1·36)	98/`69 (58·0%)	1·11 (0·91–1·20)

[*] Excludes non-US-born patients

[**] Adjusted relative risks adjusting for sex, categorical age (<1, 1-4, 5-14, 15-24, 25-34, 35-44, 45-54, 55-64, 65-74, 76-84, 85+), calendar year, and nine US Census regional divisions (Pacific, Mountain, West North Central, West South Central, East North Central, East South Central, South Atlantic, Middle Atlantic, and New England).

[a.] Includes all causes of death and all sites of TB disease.

[b.] Lung cavitation among pulmonary TB patients who had an abnormal radiograph or CT scan.

[c.] Positive sputum smear results among pulmonary TB patients who had a sputum smear test result.

Supplementary Table 9: TB treatment outcomes by race/ethnicity among US-born persons diagnosed with TB including persons with MDR-TB, United States, 2003-2018*

Race/ethnicity	Treatment discontinuation [a]	aRR** (95% CI)	Death during treatment [b]	aRR** (95% CI)
**White**	494/21,857 (2·3%)	Ref	2,428/21,857 (11·1%)	Ref
**Black**	829/28,017 (3·0%)	1·24 (1·11–1·39)	2,803/28,017 (10·0%)	1·32 (1·25–1·39)
**Hispanic**	349/12,145 (2·9%)	1·20 (1·04–1·39)	702/12,145 (5·8%)	1·19 (1·10–1·29)
**Asian**	23/2,149 (1·1%)	0·55 (0·35–0·81)	89/2,149 (4·1%)	1·13 (0·92–1·36)
**American Indian/Alaska Native**	36/2,004 (1·8%)	0·71 (0·49–0·98)	226/2,004 (11·3%)	1·45 (1·27–1·64)
**Native Hawaiian/other Pacific Islander**	8/377 (2·1%)	1·06 (0·49–1·98)	17/377 (4·5%)	1·23 (0·75–1·84)
**Other**	6/273 (2·2%)	1·02 (0·41–2·04)	22/273 (8·1%)	1·35 (0·75–1·84)

* Excludes non-US-born patients

** Adjusted relative risks adjusting for sex, categorical age (<1, 1-4, 5-14, 15-24, 25-34, 35-44, 45-54, 55-64, 65-74, 76-84, 85+), calendar year, and nine US Census regional divisions (Pacific, Mountain, West North Central, West South Central, East North Central, East South Central, South Atlantic, Middle Atlantic, and New England).

a. Includes patients who refused treatment or were lost.

b. Includes deaths that occurred at any point after diagnosis.

Supplementary Table 11: Disparities among US-born Hispanic persons by racial/ethnic subgroups 2003-2019 (n=13,056*)

Race/ethnicity	Diagnosis after death aRR**(95% CI)	Cavitation aRR** (95% CI)	Smear positive aRR**(95% CI)	Treatment > 12 months aRR** (95% CI)	Treatment discontinuation aRR** (95% CI)	Death during treatment aRR** (95% CI)
Non-Hispanic White	Ref	Ref	Ref	Ref	Ref	Ref
Hispanic White	1·41 (1·24-1·61)	0·98 (0·94-1·03	1·07 (1·05-1·10)	1·16 (1·07-1·26)	1·38 (1·20-1·58)	1·18 (1·09-1·28)
Hispanic Black	0·91 (0·42-1·69)	0·97 (0·81-1·15)	1·00 (0·88-1·13)	0·98 (0·71-1·31)	1·10 (0·59-1·85)	1·67 (1·28-2·09)
Hispanic Other*	0·84 (0·14-2·52)	1·00 (0·69-1·34)	0·99 (0·77-1·20)	1·20 (0·69-1·87)	1·13 (0·35-2·60)	1·42 (0·78-2·23)

*Of the 13,181 persons who identified as Hispanic, 125 were missing further race/ethnicity information
** Adjusted for sex, categorical age, and regional division
[a] Among pulmonary TB patients
[b] Other race/ethnicity defined as American Indian/Alaska Native, Asian, Native Hawaiian/other Pacific Islander, or Multiracial

*
